# Molecular characterization and phylogenetic analysis of dengue viruses imported into Taiwan during 2011-2016

**DOI:** 10.1371/journal.pntd.0006773

**Published:** 2018-09-20

**Authors:** Cheng-Fen Yang, Shu-Fen Chang, Tung-Chien Hsu, Chien-Ling Su, Tzy-Chen Wang, Shih-Hung Lin, Su-Lin Yang, Chien-Chou Lin, Pei-Yun Shu

**Affiliations:** Center for Diagnostics and Vaccine Development, Centers for Disease Control, Ministry of Health and Welfare, Taiwan, Republic of China; Mahidol Univ, Fac Trop Med, THAILAND

## Abstract

A total of 1,596 laboratory-confirmed imported dengue cases were identified in Taiwan during 2011–2016. Most of the imported cases arrived from Southeast Asia as well as the Indian subcontinent, the Pacific region, Latin America, Australia and Africa. Phylogenetic analyses of the complete envelope protein gene sequences from 784 imported dengue virus (DENV) isolates were conducted, and the results suggest that the DENV-1 genotype I and DENV-2 Cosmopolitan genotype comprise the predominant serotype/genotype of DENV strains circulating in Southeast Asia. The DENV-1 genotype III, DENV-3 genotype III and DENV-4 genotype I and II strains were found to be newly emerging in several Southeast Asian countries. Our results also showed that geographical restrictions of DENV-1 genotype I, DENV-1 genotype III and DENV-2 Cosmopolitan genotype are becoming blurred, indicating the extensive introductions and continuous expansions of DENV strains between nations in Southeast Asia. In this study, we present the geographic distribution and dynamic transmission of DENV strains circulating in Southeast Asian countries. In addition, we demonstrated local dengue epidemics caused by several imported DENV strains in Taiwan during 2011–2016.

## Author summary

Dengue is the most prevalent mosquito-borne viral disease in the world. The expansion of dengue viruses to different parts of the world has been accelerated by the increase in worldwide travel and trade. In this study, we present the results of a laboratory-based dengue surveillance in Taiwan during 2011–2016. A total of 1,596 laboratory-confirmed imported dengue cases were identified. The travelers were infected in 29 countries in Southeast Asia, the Indian subcontinent, the Pacific region, Latin America, Australia and Africa. Phylogenetic analyses of the envelope gene sequences of 784 imported dengue virus isolates suggest that the DENV-1 genotype I and DENV-2 Cosmopolitan genotype comprise the predominant serotype/genotype DENV strains circulating in Southeast Asia. Our results also showed that geographical restrictions of some of the DENV genotypes are becoming blurred, indicating the extensive introductions and continuous expansions of DENV strains between countries in Southeast Asia. In addition, we demonstrated dengue outbreaks in Taiwan caused by viruses imported from Asia and the Americas. The DENV envelope gene sequences from this study will contribute to a better understanding of the genetic evolution, dynamic transmission and global expansion of dengue viruses.

## Introduction

Dengue is the most prevalent mosquito-borne viral infection of humans in tropical and subtropical regions of the world [1]. In recent decades, the incidence of dengue has grown dramatically; approximately half of the world’s population is now at risk [2]. An estimated 390 million dengue infections occur annually, of which 96 million dengue infections manifest clinically [3, 4]. Dengue virus (DENV) belongs to the genus *Flavivirus* in the family *Flaviviridae*. The DENV genome consists of a single-stranded, positive-sense RNA, which is of approximately 10,700 nucleotides and contains a long open reading frame that encodes three structural proteins (capsid [C], premembrane/membrane [prM] and envelope [E] proteins) and seven nonstructural (NS) proteins (NS1, NS2A, NS2B, NS3, NS4A, NS4B, and NS5) [5, 6].There are 4 genetically and antigenically distinct DENV serotypes (DENV-1 to DENV–4) that cause dengue. DENVs are transmitted to humans through the bite of an infected female *Aedes* mosquito [7]. Dengue disease can manifest as mild dengue fever or the more severe and potentially fatal dengue hemorrhagic fever or dengue shock syndrome [8, 9].

Dengue is endemic to most countries in Southeast Asia, the Western Pacific region and the Americas, with a very high morbidity rate and disease burden [4, 10]. A large number of cases are reported each year, and all four DENV serotypes currently circulate in hyperendemic countries. The rapid expansion of DENV strains to different parts of the world has been accelerated by the increase in worldwide travel and trade. Studies on DENV infection in travelers can thus provide useful information on the geographic distribution and global movement of DENV [11–14]

Taiwan is an island off the southeastern coast of mainland China in the western Pacific Ocean. The island straddles the Tropic of Cancer, giving it a warm tropical-subtropical climate. *Aedes albopictus* is found throughout Taiwan, whereas *Ae*. *aegypti* is distributed in the south [15]. Dengue is not considered endemic in Taiwan; thus, the close commercial links and air travel between Taiwan and other countries are responsible for the constant importation of multiple DENVs and the outbreaks that occur each year [16, 17]. To reduce the introduction of DENV strains into Taiwan and prevent local epidemics, both passive and active surveillance of DENV infections have been implemented in Taiwan. We previously reported the molecular characterization of DENV strains imported into Taiwan during 2003–2010 [18, 19]. The results provided information on the geographic distribution and dynamic transmission of DENV strains in Southeast Asian countries. In this study, we continued to perform laboratory-based surveillance and provide essential information on the molecular epidemiology of DENV strains circulating in Southeast Asian countries during 2011–2016.

## Materials and methods

### Human serum samples

Dengue is a reportable infectious disease in Taiwan, and suspected cases must be reported within 24 hours of clinical diagnosis. To provide effective surveillance, both passive (the hospital-based reporting system) and active (such as fever screening at airports, self-reporting, and expanded screening for contacts of confirmed cases) surveillance systems were implemented by the central and local health departments in Taiwan. Human serum samples of suspected dengue cases were submitted to the Centers for Disease Control, Taiwan (Taiwan CDC), for confirmation of DENV infection. The human serum samples used in this study were derived from confirmed dengue cases submitted to the Taiwan CDC during 2011–2016. All samples analyzed were anonymized. The study protocol was reviewed and approved by the Taiwan CDC Institutional Review Board (IRB 104121). The informed consent requirement was waived by the board. An imported dengue case was defined as a laboratory-confirmed dengue case with travel history to endemic countries within 14 days before the date of onset of dengue. An indigenous case was recorded when no overseas travel was indicated.

### Laboratory diagnosis

DENV infection was defined as a febrile illness associated with the detection of DENV RNA by reverse transcription-polymerase chain reaction (RT-PCR), isolation of DENV by cell culture, detection of DENV nonstructural protein 1 (NS1) antigen, or a seroconversion or at least a four-fold increase in the titer of IgM or IgG antibodies against DENV in paired acute and convalescent serum samples tested by capture IgM and IgG enzyme-linked immunosorbent assays (ELISA). Isolation of DENV was performed using a mosquito cell line (clone C6/36 of *Ae*. *albopictus* cells) as previously described [18]. Briefly, for each acute-phase serum sample, 50 μL of the sample diluted at ratios of 1:20, 1:40, 1:80, and 1:160 with RPMI 1640 medium (Gibco/BRL, Life Technologies, Auckland, New Zealand) containing 1% fetal calf serum was added to a 96-well microtiter plate. Then, 1x10^5^ cells/100 μL/well of C6/36 were added to the microtiter plate and incubated for 7 days at 28°C. Cells were harvested, and infection was confirmed by immunofluorescence assay using dengue serotype-specific monoclonal antibodies, including 5F3-1 (DENV-1 specific, ATCC HB-47), 3H5-1 (DENV-2 specific, ATCC HB-46), 5D4-11 (DENV-3-specific, ATCC HB-49) and 1H10-6 (DENV-4-specific, ATCC HB-48). The viruses were subcultured in C6/36 cells and harvested for nucleotide sequencing after the first or second passage. Isolated viruses were identified using the nomenclature of serotype/country of origin/strain/year of isolation. To detect and differentiate DENV serotypes in acute-phase samples, we performed one-step, SYBR Green I-based, real-time RT-PCR (QuantiTect SYBR Green RT-PCR kit, Qiagen, Hilden, Germany) using the LightCycler 96 Real-Time PCR System (Roche Diagnostics, Mannheim Germany). Real-time RT-PCR was performed using two sets of consensus primers, one primer set targeting a region of the nonstructural protein 5 (NS5) genes to detect all of the flaviviruses and the other primer set targeting a region of the C gene to detect all of the DENV serotypes. The DENV serotypes of the positive samples were then confirmed by DENV serotyping using four sets of serotype-specific primers targeting the C gene [20]. A commercial DENV NS1 Ag strip rapid test kit (Bio-Rad Laboratories, Marnes La Coquette, France) and SD Dengue NS1 Ag test (Standard Diagnostics, Inc. Kyonggi-do, Korea) were used to detect the DENV NS1 antigen in serum samples. Envelope (E)/Membrane (M)-specific capture IgM and IgG ELISA were used to detect DENV-specific IgM and IgG antibodies as previously described [21].

### Preparation of viral RNA, RT-PCR amplification and nucleotide sequencing

Viral RNA was extracted from acute-phase serum samples or the culture supernatant of C6/36 cells infected with each of the isolated DENV strains using a QIAamp Viral RNA Mini Kit (QIAGEN, Hilden, Germany). Primers used for amplification and sequencing of C, prM and E gene sequences of DENVs were described previously [17] [18]. The RT-PCR reaction was carried out with the SuperScript III One-Step RT-PCR system with Platinum Taq High Fidelity (Invitrogen). The cDNA synthesis step was performed at 55°C for 30 min; PCR at 94°C for 2 min; 40 cycles of 94°C for 15 sec, 50°C for 30 sec, and 68°C for 1 min; and prolonged elongation at 68°C for 5 min. PCR products were purified using a Qiagen QIA quick Gel Extraction Kit (QIAGEN). Nucleotide sequences were determined by an automated DNA sequencing kit and an ABI Prism 3730XL DNA sequencer (Applied Biosystems, Foster City, CA) according to the manufacturer’s protocols. Overlapping nucleotide sequences were combined for analysis and edited with the Lasergene software package (DNASTAR Inc, Madison, WI). Nucleotide sequences of the complete E gene of the DENV strains described in this study were submitted to GenBank with the following accession numbers: 334 DENV-1 strains (KT175076-KT175078, KT175082-KT175101, KT175103-KT175110, KU365900, KY496854, KY496855, and MG894671-MG894970), 234 DENV-2 strains (KT175111-KT175140, KU365901, and MG894971-MG895173), 133 DENV-3 strains (KP176703-KP176710, KP175715, MG895174-MG895297), and 99 DENV-4 strains (MG895298-MG895396). All the strain identifiers and their accession numbers are shown in the S1 Table.

### Phylogenetic analysis

The nucleotide sequences of the complete E gene of 784 imported and 16 epidemic strains in Taiwan in combination with sequences of epidemic strains from Southeast Asian countries and various global reference strains of different genotypes available from GenBank were analyzed. In addition, sequences representing the most closely related to the epidemic strains in Taiwan obtained using BLAST were selected for phylogenetic analyses. Sequences of DENV strains were aligned, edited and analyzed using Clustal W software [22]. The phylogenetic analysis was performed using MEGA version 7 (http://www.megasoftware.net/) [23]. To construct the phylogenetic trees, the maximum likelihood method using the general time reversible as a substitution model and the neighbor-joining method using the maximum composite likelihood as a substitution model were utilized. The reliability of the analysis was evaluated by a bootstrap test with 1,000 replications. Sequences of D2/New Guinea/NGC/1944 strain/M29095, D2/Senegal/DAKHD10674/1970/AF231720, D1/USA/Hawaii/1945 strain/AF425619 and D2/New Guinea/NGC/1944 strain/M29095, were used as outgroups to root the tree of the DENV-1, DENV-2, DENV-3 and DENV-4 strains, respectively.

## Results

### Imported dengue cases in Taiwan during 2011–2016

**Fig 1:**
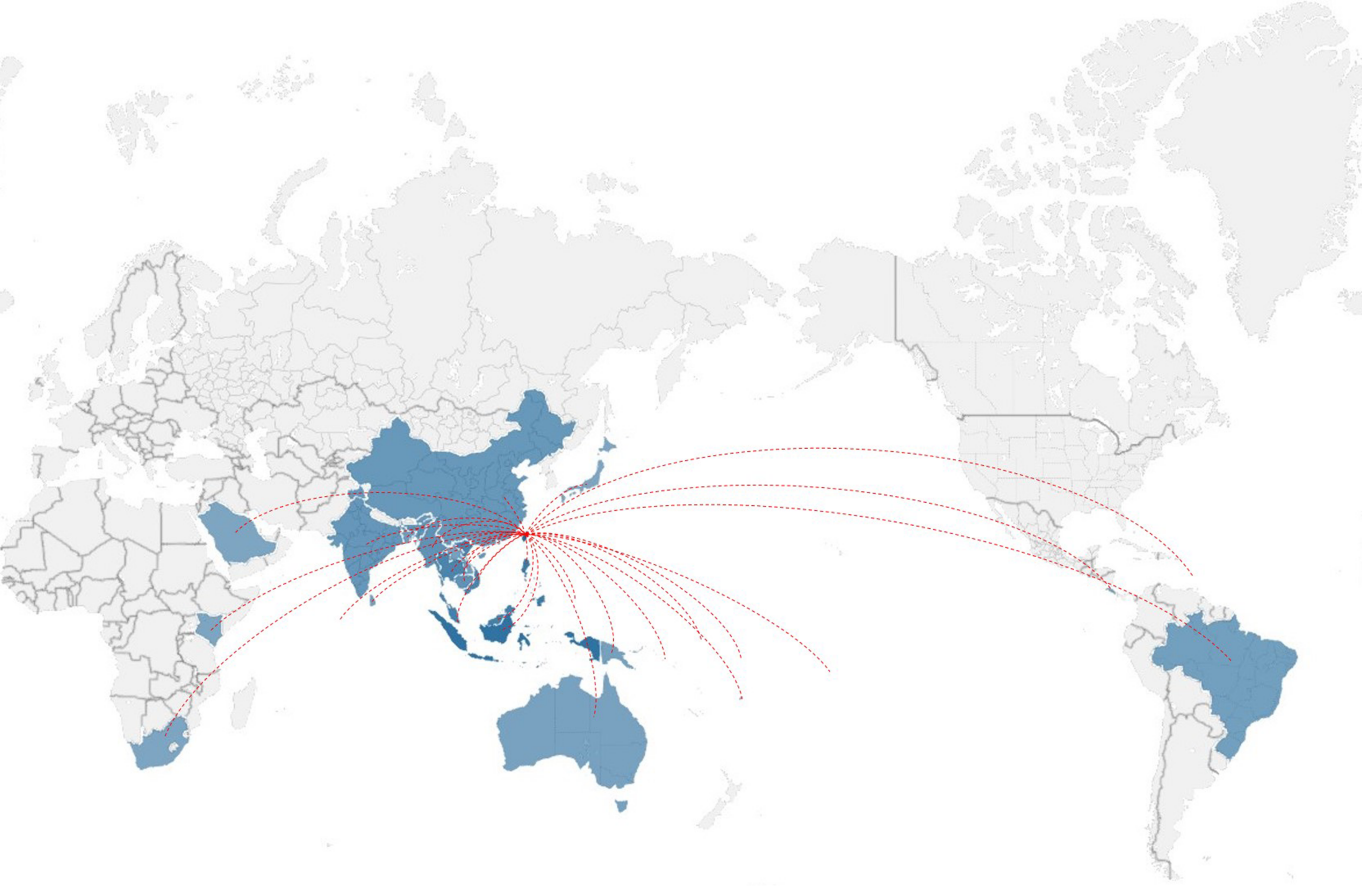
Map showing dengue cases imported from 29 countries into Taiwan during 2011–2016 (map created using Power map within Microsoft Excel).

**Fig 2:**
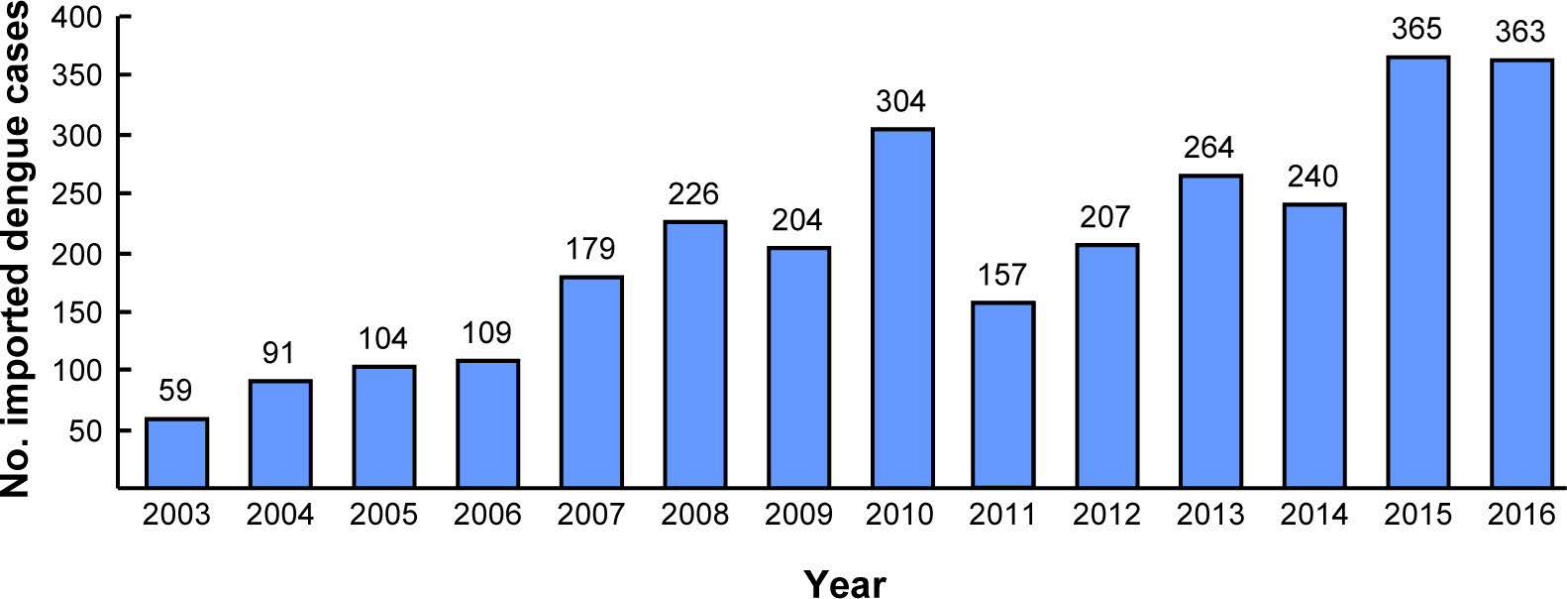
Number of imported dengue cases in Taiwan between 2011 and 2016.

A total of 1,596 laboratory-confirmed imported dengue cases (both visitors to Taiwan and local returning residents) were identified in Taiwan during 2011–2016. Among them, 703 cases (44.0%) were identified by fever screening at airports (Table 1) and most (>90%) of these cases were in their viremic stages with positive real-time RT-PCR and negative IgM and IgG results. Most cases arrived from Southeast Asian countries, with Indonesia (24.8%, 396 cases), the Philippines (19.2%, 306 cases), Malaysia (14.2%, 226 cases), Thailand (12.0%, 192 cases), and Vietnam (12.0%, 191 cases) being the most frequent country sources of importation. Cases were also imported from other Asian countries (16.6%, 265 cases, including Myanmar, Singapore, Cambodia, India, China, Bangladesh, Maldives, Sri Lanka, Laos, Saudi Arabia, and Japan), the Pacific region (0.7%, 11 cases, including Palau, Papua New Guinea, Nauru, Fiji, Solomon Islands, Tuvalu, and French Polynesia), Australia (0.1%, 2 cases), Latin America (0.4%, 7 cases, including Brazil, Costa Rica, and Saint Lucia), and Africa (0.1%, 2 cases, one from South Africa and the other from Kenya). Comparing the numbers of imported dengue cases between 2003–2010 and 2011–2016, we found there is an increasing trend of imported cases from the Philippines, Malaysia and Singapore during the study period. Fig 1 shows the country sources of importation of DENVs in Taiwan during 2011–2016. Fig 2 shows the number of imported dengue cases in Taiwan during 2003–2016.

**Table 1:** Summary of serotype and genotype distributions of DENV strains obtained from imported cases in Taiwan during 2011–2016.

Country origin/Serotype	Imported case	Imported case identified by fever screening at airports	DENV-1				DENV-2			DENV-3				DENV-4			Un- serotyped
Genotype			I	II	III	Un-genotyped	Asian 1	Cosmopolitan	Un-genotyped	I	II	III	Un-genotyped	I	II	Un-genotyped	
Indonesia	393	196	57	8	0	9	0	56	21	63	0	0	23	0	15	3	138
Philippines	306	112	1	47	0	4	0	33	12	25	0	0	4	5	36	5	134
Malaysia	226	132	66	2	11	6	2	40	10	2	0	11	3	1	6	1	65
Thailand	192	78	24	0	1	3	22	7	11	0	3	13	5	9	0	2	92
Vietnam	191	75	48	0	1	8	14	7	15	0	3	1	0	11	0	2	81
Myanmar	61	18	13	0	0	3	8	0	2	0	0	0	1	6	0	1	27
Singapore	58	35	2	0	13	4	0	16	1	1	0	1	1	0	1	1	17
Cambodia	53	15	10	0	0	4	3	0	3	0	1	0	2	4	0	1	25
India	32	18	0	0	6	2	0	9	0	0	0	4	1	0	0	0	10
China	29	7	7	0	3	3	0	2	1	0	0	0	0	0	0	0	13
Maldives	10	1	0	0	2	0	0	3	0	0	0	0	0	1	0	0	4
Bangladesh	10	5	0	0	3	1	0	0	0	0	0	0	1	0	0	0	5
Sri Lanka	6	0	2	0	0	0	0	0	0	0	0	0	0	0	0	0	4
Brazil	5	2	0	0	0	3	0	0	0	0	0	0	0	0	1	0	1
Laos	4	1	1	0	0	0	1	0	0	0	1	0	0	0	0	0	1
Papua New Guinea	4	1	0	0	0	1	0	0	0	0	0	0	0	0	1	0	2
Australia	3	2	0	0	0	0	0	0	0	0	0	0	0	0	0	0	3
Nauru	2	0	0	0	0	0	0	0	0	0	0	0	0	0	0	0	2
Japan	1	0	0	0	0	0	0	0	0	0	0	0	0	0	0	0	1
Saudi Arabia	1	0	0	0	0	0	0	1	0	0	0	0	0	0	0	0	0
Solomon Islands	1	0	0	0	0	0	0	0	0	1	0	0	0	0	0	0	0
Fiji	1	0	0	0	0	0	0	0	0	0	0	0	0	0	0	0	1
French Polynesia	1	0	0	0	0	0	0	0	0	0	0	0	0	0	0	0	1
Tuvalu	1	1	0	0	0	0	0	1	0	0	0	0	0	0	0	0	0
Palau	1	1	0	0	0	0	0	1	0	0	0	0	0	0	0	0	0
Costa Rica	1	1	0	0	1	0	0	0	0	0	0	0	0	0	0	0	0
Saint Lucia	1	1	0	0	0	0	0	0	0	0	0	0	0	0	1	0	0
Kenya	1	1	0	0	0	0	0	1	0	0	0	0	0	0	0	0	0
South Africa	1	0	0	0	0	0	0	0	0	0	0	0	0	0	0	0	1
Total	1596	703	231	57	41	51	50	177	76	92	8	30	41	37	61	16	628

Un-serotyped: imported dengue case with negative RT-PCR and positive IgM and IgG results.

### Serotype distributions of imported DENV strains

**Fig 3:**
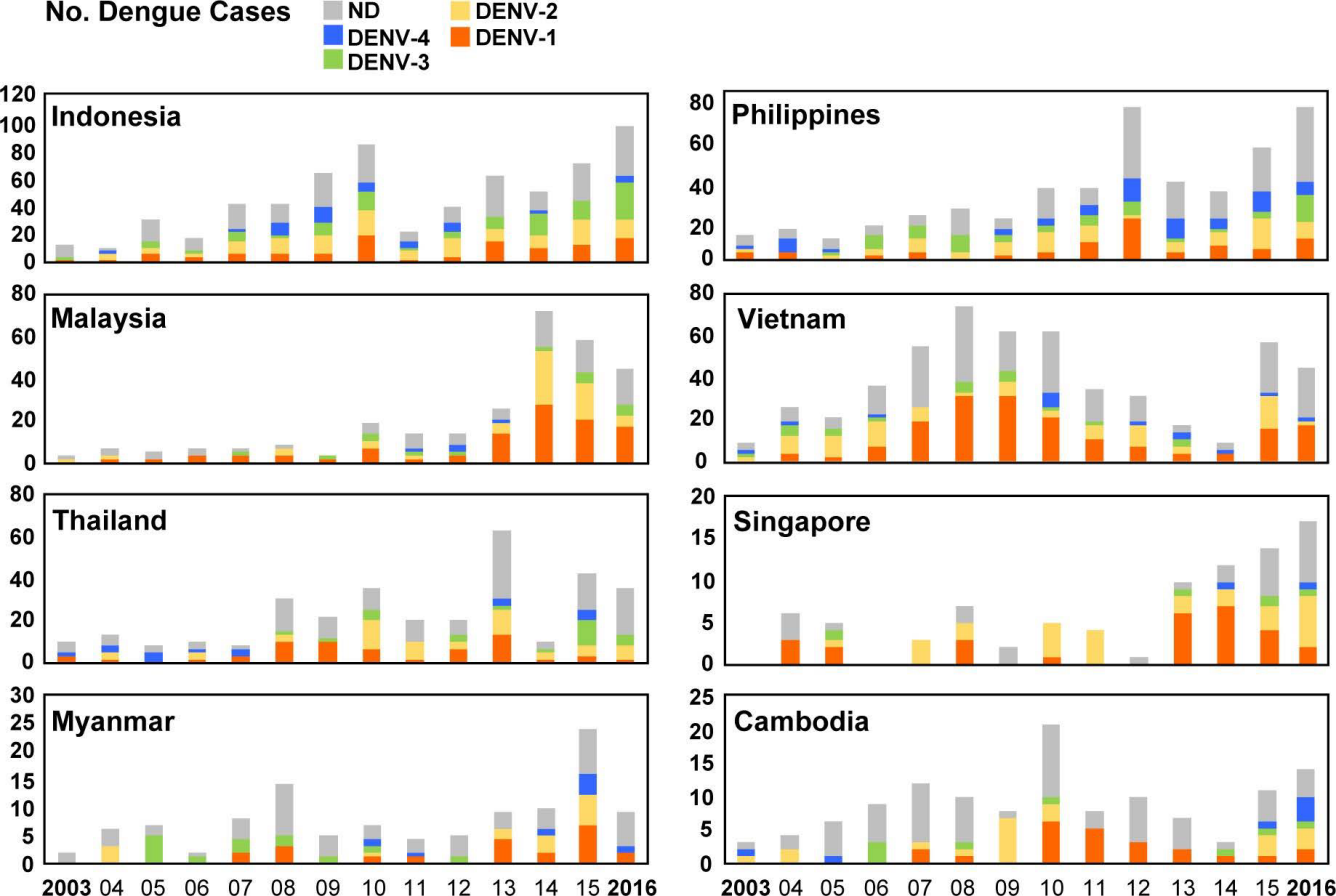
The serotype distributions of the DENV strains imported into Taiwan during 2003–2016. The DENV strains imported from the eight most common countries are summarized to show the changes in circulating serotypes in each country. ND: not determined.

From the 1,596 imported dengue cases, 380, 303, 171 and 114 cases were determined to be infected with DENV-1, DENV-2, DENV-3, and DENV-4, respectively. Table 1 summarizes serotype and genotype distributions of imported DENV strains from 29 countries. The serotype distributions of DENV strains imported from the most common Southeast Asian countries each year during 2003–2016 are shown in Fig 3. Yearly changes in serotype distribution were observed, and all four serotypes of DENV were found to circulate in each of these countries during 2011–2016. The number of imported dengue cases from Malaysia increased sharply during 2014–2016, and the main serotypes were DENV-1 and DENV-2. The main serotype of imported DENV strains from Vietnam shifted from DENV-1 during 2007–2010 to DENV-2 during 2012 and 2015 and then back to DENV-1 during 2016. The number of imported dengue cases from Singapore increased significantly during 2013–2016, and the main serotypes in recent years have been DENV-1 and DENV-2. A relatively high number of imported cases was observed from Myanmar in 2015, and DENV-1, DENV-2 and DENV-4 were the main serotypes. All 4 serotypes of DENV were found to cocirculate in Cambodia during 2015–2016.

### Genotype distributions of imported DENV strains

**Fig 4:**
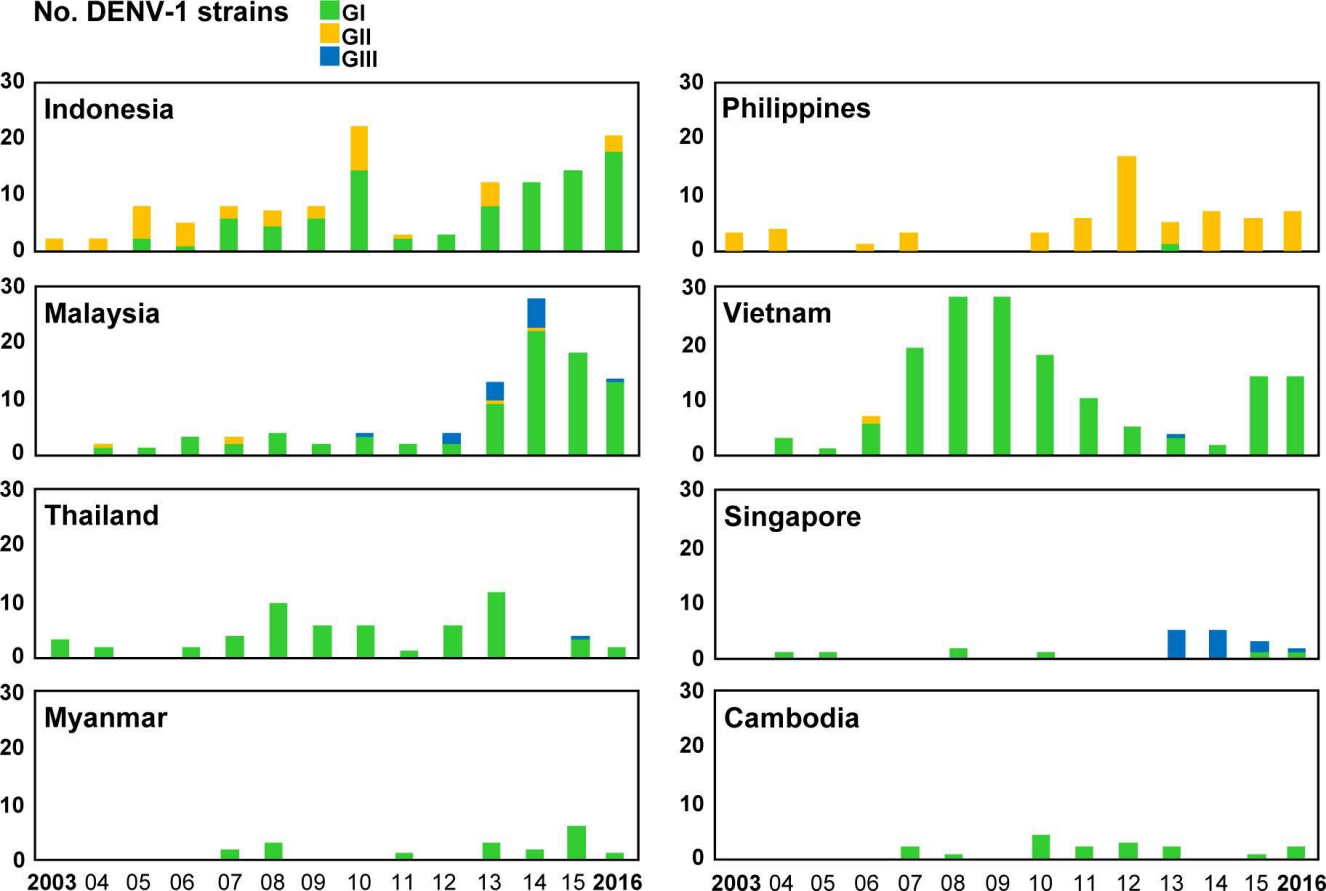
The genotype distributions of the DENV-1 strains imported into Taiwan during 2003–2016. The DENV strains imported from the eight most common countries are summarized to show the changes in circulating genotypes in each country. GI: genotype I; GII: genotype II; GIII: genotype III.

**Fig 5:**
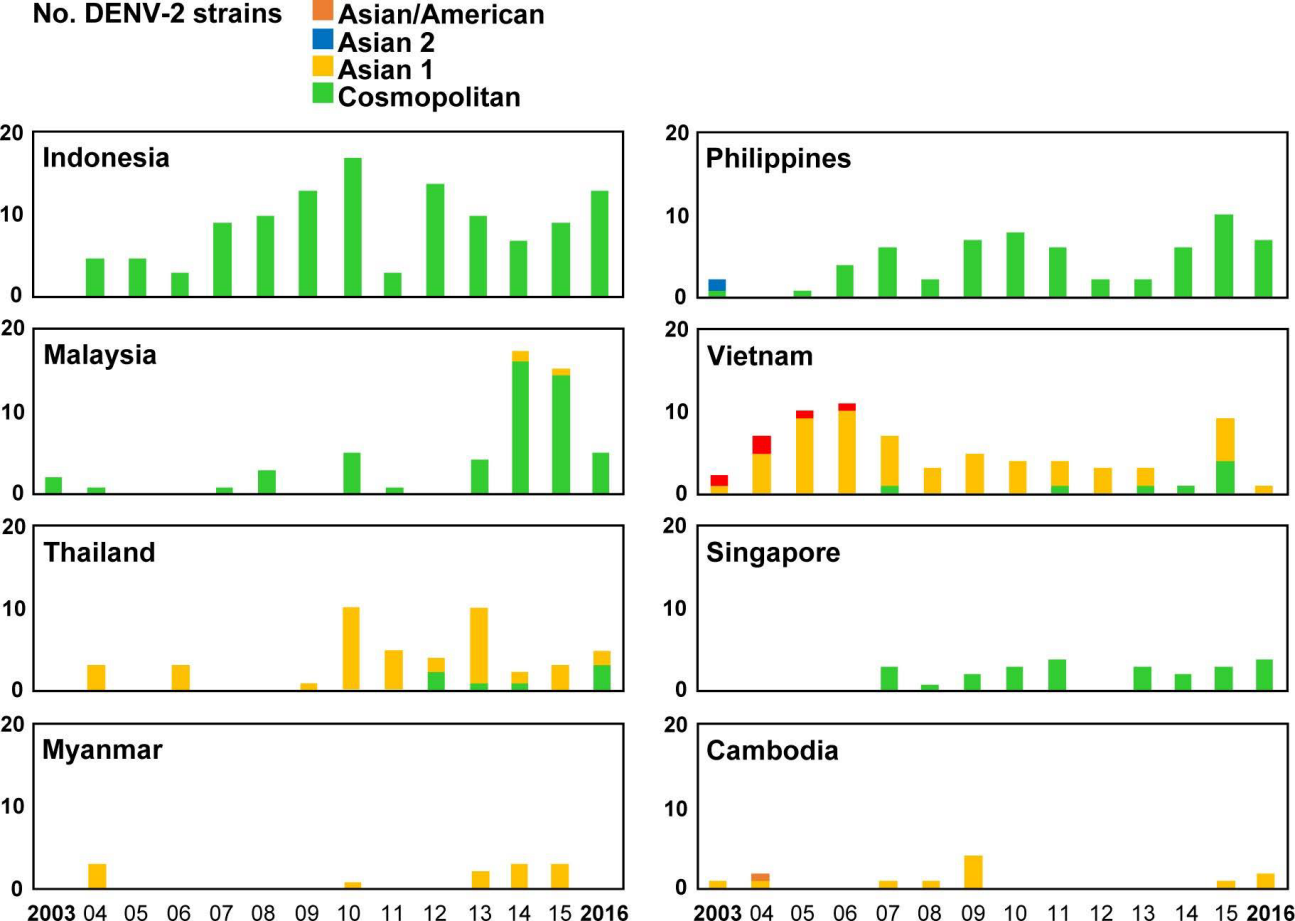
The genotype distributions of the DENV-2 strains imported into Taiwan during 2003–2016. Asian/American: Asian/American genotype; Asian 2: Asian genotype 2; Asian 1: Asian genotype 1; Cosmopolitan: cosmopolitan genotype. See the legend of Fig 3 for other details.

**Fig 6:**
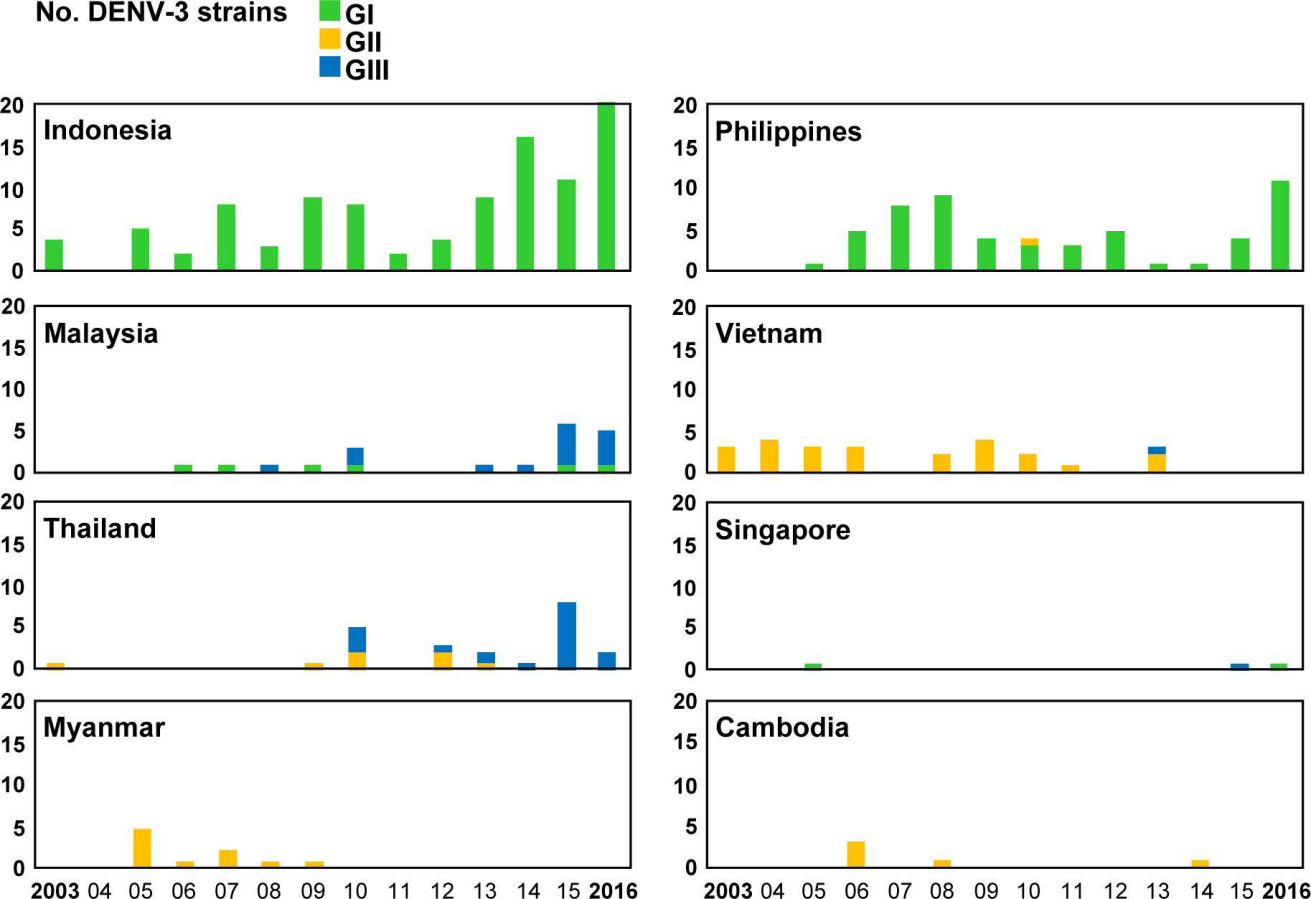
The genotype distributions of the DENV-3 strains imported into Taiwan during 2003–2016. GI: genotype I; GII: genotype II; GIII: genotype III. See the legend of Fig 3 for other details.

**Fig 7:**
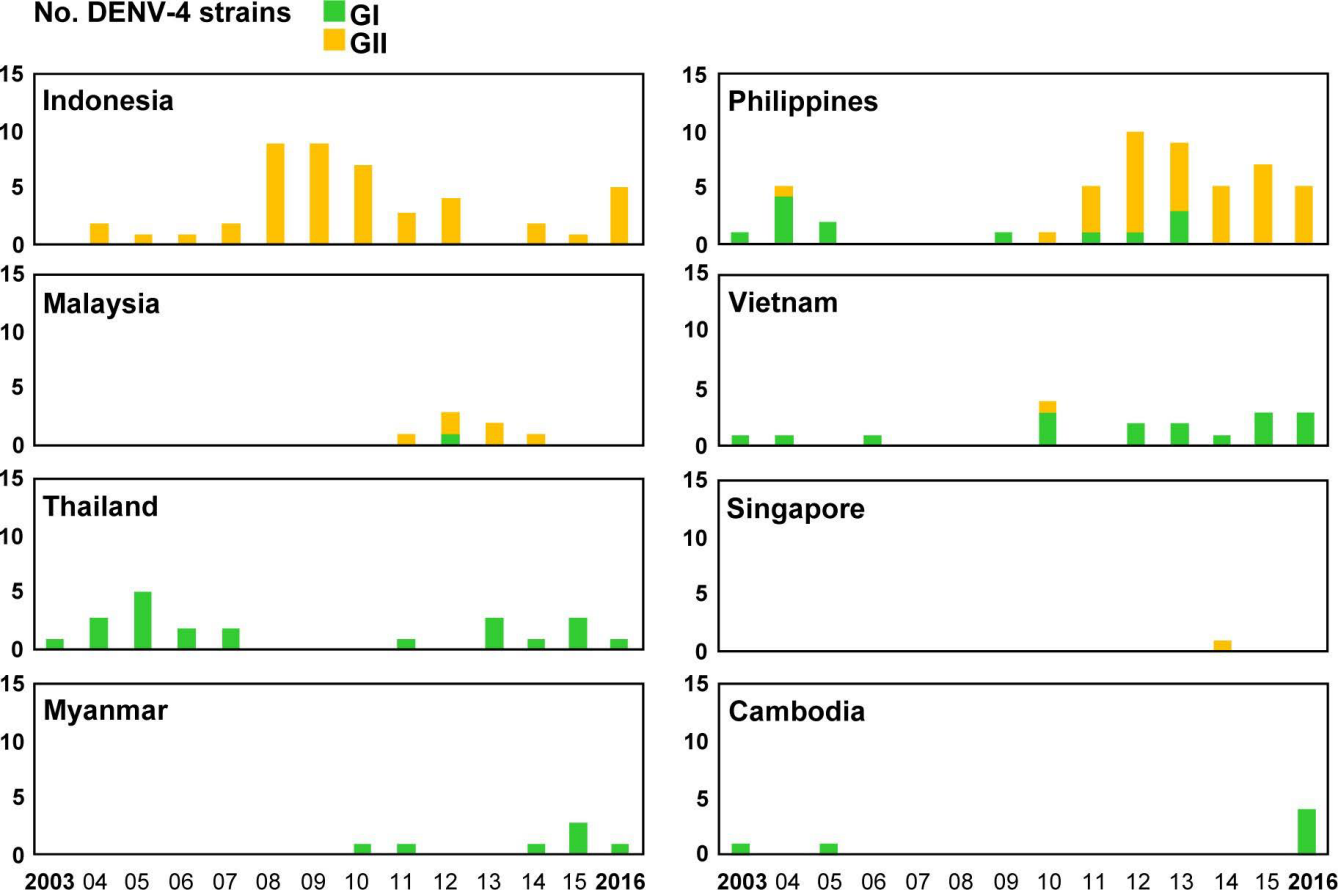
The genotype distributions of the DENV-4 strains imported into Taiwan during 2003–2016. GI: genotype I; GII: genotype II. See the legend of Fig 3 for other details.

Among the 1,596 imported dengue cases, 784 DENV strains were isolated from acute-phase serum samples of patients infected in 23 countries (Table 1). Phylogenetic analyses of the E gene sequences of imported DENV strains were conducted to determine the genotype and genetic relationship of these viral strains. The designations of DENV genotypes are based on the classification of A-Nuegoonpipat et al. [24], Twiddy et al. [25], Lanciotti et al. [26], and Klunthong et al. [27] for the DENV-1, DENV-2, DENV-3 and DENV-4 strains, respectively. The genotype distributions of the DENV-1 to DENV-4 strains imported from the 8 most common Southeast Asian countries during 2003–2016 are shown in Figs 4–7, respectively.

Fig 4 shows genotype distributions of imported DENV-1 strains. The DENV-1 strains obtained from Asia and the Pacific can be classified into three genotypes (I, II and III). Genotype I contains the majority of strains from Asia, while genotype II comprises a smaller set of Asian and Pacific strains. Genotype III contains viruses from a wide geographic area [24]. Genotype I of DENV-1 was the predominant genotype imported from Southeast Asian countries, including Indonesia, Malaysia, Vietnam, Thailand, Myanmar and Cambodia. Before 2006, genotype II of imported DENV-1 was the main genotype in Indonesia; however, since 2007, the main genotype has shifted to genotype I. Genotype II was the predominant genotype of imported DENV-1 from the Philippines. The numbers of genotype III of imported DENV-1 strains from Malaysia and Singapore increased during 2013–2014.

Fig 5 shows genotype distributions of imported DENV-2 strains. The DENV-2 strains can be classified into six genotypes. The Cosmopolitan genotype has a wide geographic distribution. The Asian genotype 1 and 2 contain viruses from Asia, and the Asian/American genotype comprises viruses from Southeast Asia and Latin America. The American genotype consists of viruses from Latin America and older isolates collected from Indian subcontinent and the Pacific, and the Sylvatic genotype contains sylvatic strains from Asia and Africa [25]. The Cosmopolitan genotype was the predominant genotype of imported DENV-2 strains from Indonesia, the Philippines, Malaysia, and Singapore, and Asian genotype 1 was the main genotype of imported DENV-2 strains from Vietnam, Thailand, Myanmar and Cambodia.

Fig 6 shows genotype distributions of imported DENV-3 strains. The DENV-3 strains can be classified into four genotypes. Genotype I consists of viruses from Indonesia, Malaysia, the Philippines, the Pacific islands and Australia. Genotype II contains viruses from Southeast Asia. Genotype III has a wide geographical distribution which includes Asia, Africa and Latin America. Genotype IV consists of viruses from Puerto Rico and the 1965 Tahiti virus isolates [26]. Genotype I was the predominant genotype of imported DENV-3 strains from Indonesia and the Philippines. In Malaysia, the number of imported genotype III strains increased between 2015 and 2016. Genotype II was the main genotype of imported DENV from Vietnam, Myanmar and Cambodia. In Thailand, the main detected genotype has shifted from genotype II to genotype III in recent years.

Fig 7 shows genotype distributions of imported DENV-4 strains. The DENV-4 strains were separated into four genotypes. Genotype I contains viruses from Asia. Genotype II consists of viruses from Asia, the Pacific and Latin America. Genotype III contains viruses from Thailand and genotype IV contains sylvatic strains from Malaysia [27]. Genotype I was the predominant genotype of imported DENV-4 from Vietnam, Thailand, Myanmar and Cambodia, whereas Genotype II was the main genotype from Indonesia and Malaysia. In the Philippines, the main detected genotype shifted from genotype I during 2003–2009 to genotype II during 2010–2016.

**Fig 8:**
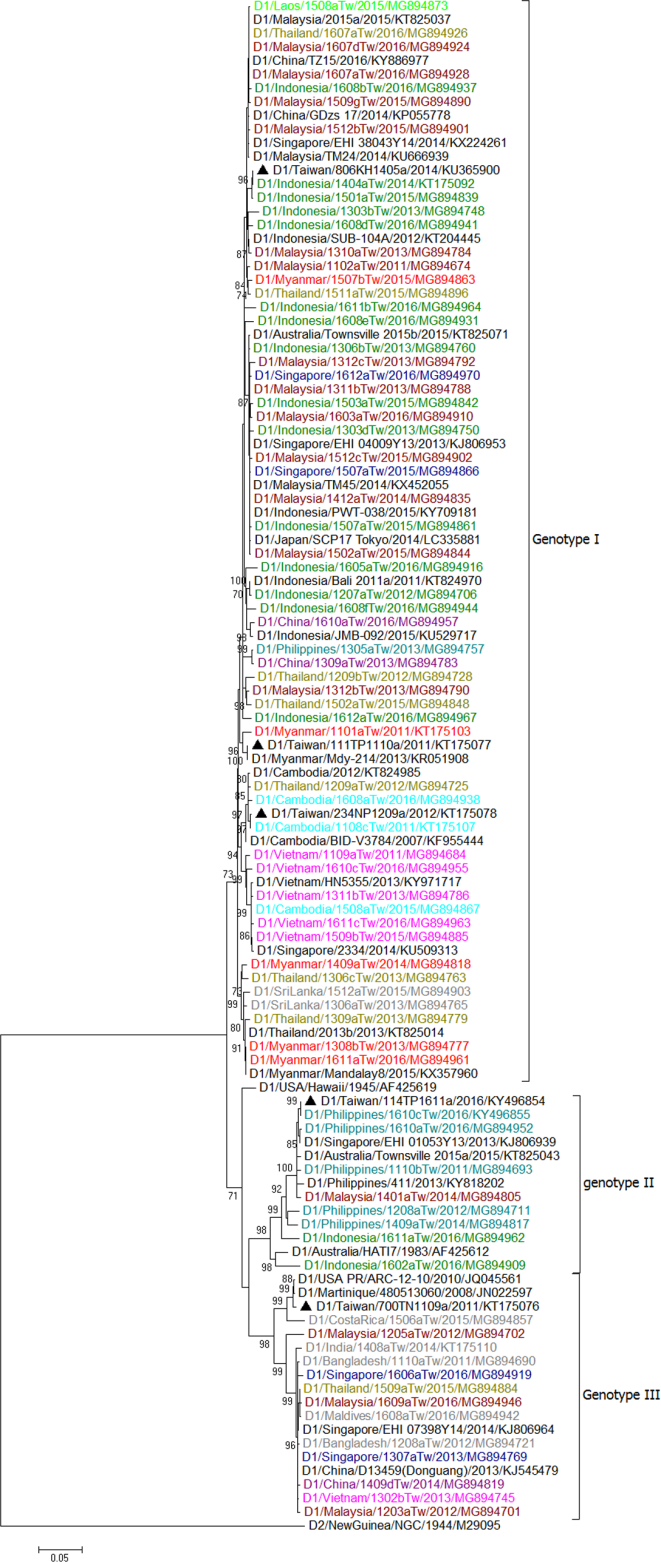
A phylogenetic tree of dengue virus type 1 (DENV-1). The phylogenetic tree is based on the complete E gene sequences of DENV-1 strains from imported and indigenous dengue cases in Taiwan during 2011–2016. The tree was constructed by the maximum likelihood method and the general time reversible model. Bootstrap support values greater than 70 are shown. Viruses were identified by using the nomenclature of serotype/country/strain/year of isolation/GenBank accession number. The Taiwan isolates from imported cases are colored by country sources of importation, with Malaysia in dark red, Indonesia in green, Singapore in navy blue, China in purple, Thailand in deep yellow, the Philippines in blue green, Myanmar in red, Vietnam in peach, Cambodia in sky blue, Laos in light green and others in grey color. The Taiwan isolates from indigenous cases are designated in black triangle. DENV reference strains retrieved from GenBank are shown in black color. The scale bar on the left indicates substitutions per site.

**Fig 9:**
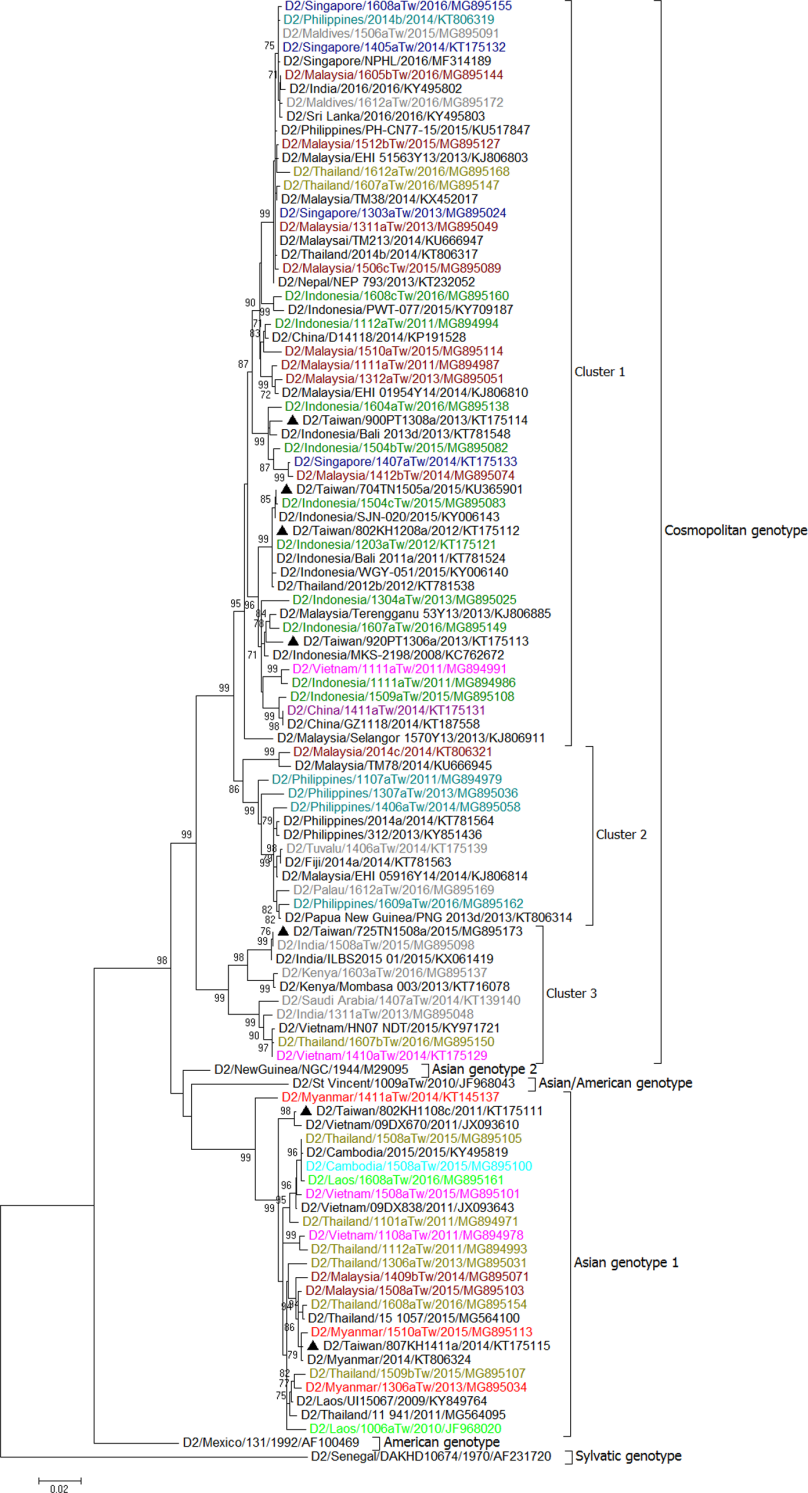
A phylogenetic tree of dengue virus type 2 (DENV-2). The phylogenetic tree is based on the complete E gene sequences of DENV-2 strains from imported and indigenous dengue cases in Taiwan during 2011–2016. See the legend of Fig 8 for other details.

**Fig 10:**
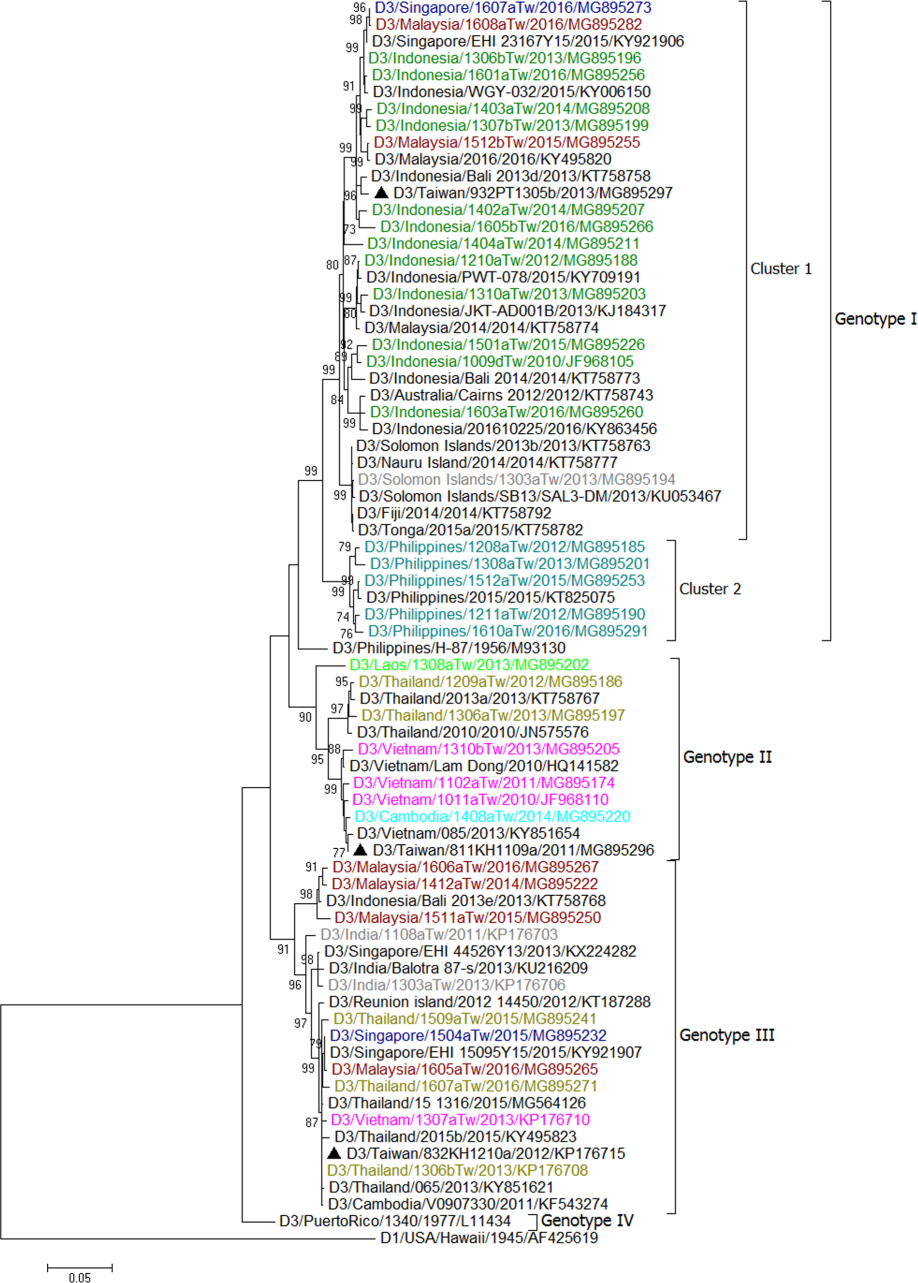
A phylogenetic tree of dengue virus type 3 (DENV-3). The phylogenetic tree is based on the complete E gene sequences of DENV-3 strains from imported and indigenous dengue cases in Taiwan during 2011–2016. See the legend of Fig 8 for other details.

**Fig 11:**
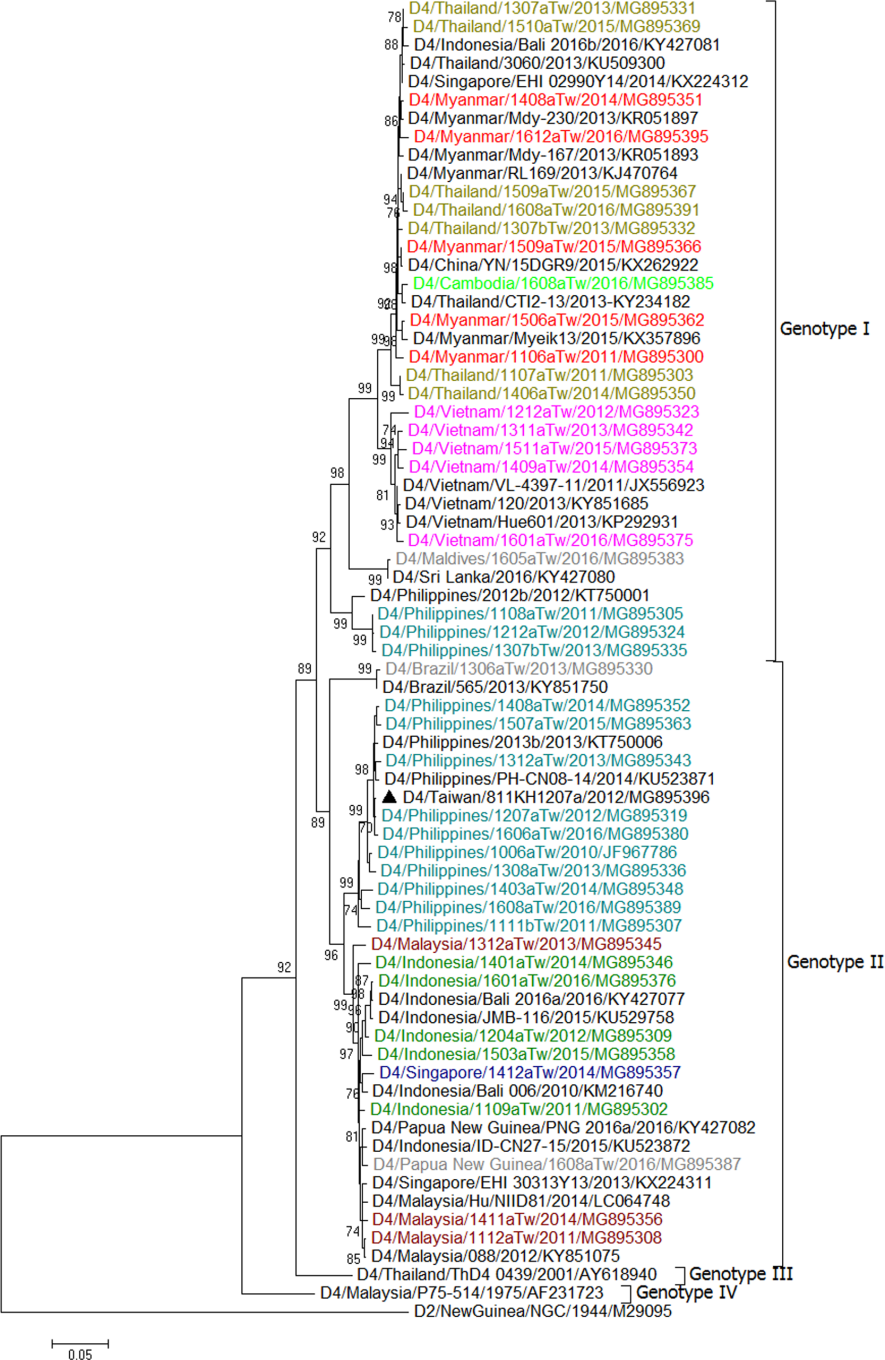
A phylogenetic tree of dengue virus type 4 (DENV-4). The phylogenetic tree is based on the complete E gene sequences of DENV-4 strains from imported and indigenous dengue cases in Taiwan during 2011–2016. See the legend of Fig 8 for other details.

We first made trees for all E gene sequences of the imported and epidemic strains in Taiwan in combination with sequences of epidemic strains from Southeast Asian countries and various global reference strains of different genotypes available from GenBank. In addition, sequences representing the most closely related to the epidemic strains in Taiwan obtained using BLAST were selected for phylogenetic analyses. The results are shown in S1 Fig–S4 Fig for DENV-1 to DENV-4, respectively. The representative E gene sequences based on country source of importation and date of sample collection, were selected to build the trees in Figs 8–11.

### Phylogenetic tree of imported DENV-1 strains

Except for the Philippines, most of the DENV-1 strains isolated from imported cases from Southeast Asian countries belonged to genotype I (Table 1 and Fig 8). Imported DENV-1 genotype I strains from Indonesia and Malaysia showed a high degree of genetic diversity and strains in different lineages that were co-circulating in these countries. Some of the strains from Thailand, Singapore, Myanmar, Laos and China were clustered with strains from Indonesia and Malaysia. Viral strains from Cambodia were closely related to viruses from Vietnam and Thailand, whereas viral strains from Myanmar and Sri Lanka were clustered with viruses from Thailand. Genotype II contained imported viral strains from the Philippines, Malaysia and Indonesia. Genotype III contained imported viral strains from diverse geographical regions, including Asia (Singapore, Bangladesh, Malaysia, Maldives, Thailand, China and India) and the Americas (USA and Costa Rica). It is interesting to note that the strains for DENV-1 tend to be less geographically clustered than in the other serotypes.

### Phylogenetic tree of imported DENV-2 strains

The DENV-2 strains isolated from imported cases during 2011–2016 fell into two genotypes, the Cosmopolitan genotype and Asian genotype 1 (Table 1 and Fig 9). The Cosmopolitan genotype strains from imported cases can be divided into three clusters. Cluster 1 contains viral strains from Malaysia, Singapore and Indonesia. Some of the imported viral strains from Thailand, Maldives, Vietnam and China also fell into this cluster. Cluster 2 contains imported viral strains from the Philippines, Tuvalu and Palau. Cluster 3 contains imported strains from India, Saudi Arabia and Kenya. A strain from Thailand and two strains from Vietnam were also found to cluster with strains from India. Asian genotype 1 contains viral strains from Thailand, Vietnam, Cambodia, Lao, and Myanmar. Imported strains from Malaysia also fell into this genotype. No Asian/American genotype and Asian genotype 2 strains were found among imported cases during 2011–2016.

### Phylogenetic tree of imported DENV-3 strains

The DENV-3 strains isolated from imported cases fell into three genotypes, genotype I, II and III (Table 1 and Fig 10). Genotype I can be divided into two clusters: one contains viral strains from Indonesia, Malaysia, Singapore and Solomon Islands, and the other contains viral strains from the Philippines. Genotype II contains imported strains from Vietnam, Thailand, Cambodia, and Laos. Genotype III contains viral strains from diverse geographical localities, including India, Singapore, Malaysia, Thailand, Vietnam and Cambodia.

### Phylogenetic tree of imported DENV-4 strains

The DENV-4 strains isolated from imported cases fell into two genotypes, genotype I and II (Table 1 and Fig 11). Genotype I contains two major clusters: one cluster contains viral strains from the Philippines, and the other contains viral strains from Vietnam, Thailand, Myanmar and Cambodia. In 2016, an imported strain from the Maldives also fell into this cluster and was closely related to virus strains from Sri Lanka. Genotype II contains imported viral strains from the Philippines, Indonesia, Malaysia and Singapore. Imported viral strains from Papua New Guinea and Brazil also belonged to this genotype.

### Major dengue epidemics in Taiwan during 2011–2016

Table 2 lists the major dengue outbreaks and epidemic DENV strains circulating in Taiwan during 2011–2016. Our results showed that a DENV-1 strain (D1/Taiwan/700TN1109a/2011) caused outbreaks in southern Taiwan during 2011–2013. This strain belongs to genotype III of DENV-1 and is closely related to viral strains from the Americas. This is the first time that an American DENV strain caused an epidemic in Taiwan. In 2011, the other 3 epidemic strains, DENV-1 (D1/Taiwan/111TP1110a/2011), DENV-2 (D2/Taiwan/802KH1108c/2011) and DENV-3 (D3/Taiwan/811KH1109a/2011), caused outbreaks in Taipei City, Kaohsiung City and Penghu County. These strains were likely introduced from Myanmar and Vietnam. In 2012, in addition to the epidemic strain D1/Taiwan/700TN1109a/2011, the other four DENV strains (D1/Taiwan/234NP1209a/2012, D2/Taiwan/802KH1208a/2012, D3/Taiwan/832KH1210a/2012 and D4/Taiwan/811KH1207a/2012) caused outbreaks in New Taipei City and Kaohsiung City. These strains were likely introduced from Cambodia, Indonesia, Thailand and the Philippines. In 2013, in addition to the epidemic strain D1/Taiwan/700TN1109a/2011, which was transmitted to Pingtung County, the other three strains (D2/Taiwan/920PT1306a/2013, D2/Taiwan/900PT1308a/2013 and D3/Taiwan/932PT1305b/2013) caused outbreaks in southern Taiwan. These strains were likely introduced from Indonesia. During 2014–2015, there was a large outbreak caused by a DENV-1 strain (D1/Taiwan/806KH1405a/2014) in southern Taiwan; this epidemic strain belonged to genotype I and is closely related to virus strains from Indonesia. In 2014, a DENV-2 strain (D2/Taiwan/807KH1411a/2014) also caused a small outbreak in Kaohsiung City. In 2015, a DENV-2 strain (D2/Taiwan/704TN1505a/2015) caused a large outbreak in Tainan City and later in Kaohsiung City, this strain belonged to the Cosmopolitan genotype and is closely related to strains from Indonesia. From January to April 2016, a total of 372 indigenous cases were identified. These cases represented the last wave of the 2015 outbreak in southern Taiwan. In 2016, there were only 8 indigenous cases identified between May and December in Taiwan. A DENV-1 strain (D1/Taiwan/114TP1611a/2016) caused a small outbreak in Taipei City in November 2016; this strain belonged to Genotype II and is closely related to strains from the Philippines. (Figs 8–11)

**Table 2:** Major dengue epidemics in Taiwan between 2011 and 2016.

Year	Epidemic area	Epidemic DENV Serotype	Epidemic DENV Genotype	Epidemic DENV strain/GenBank accession number	Possible source of epidemic virus	Estimated no. of confirmed indigenous cases	Total no. of confirmed indigenous cases	Total no. of confirmed imported cases
2011	Tainan City & Kaohsiung City	DENV-1	III	**D1/Taiwan/700TN1109a/2011/KT175076**^**a**^	Central America	~95	1545	157
	Taipei City	DENV-1	I	D1/Taiwan/111TP1110a/2011/KT175077	Myanmar	20		
	Kaohsiung City & Penghu County	DENV-2	Asian 1	D2/Taiwan/802KH1108c/2011/KT175111	Vietnam	~1266		
	Kaohsiung City	DENV-3	II	D3/Taiwan/811KH1109a/2011/MG895296	Vietnam	~149		
2012	Tainan City and Kaohsiung City	DENV-1	III	**D1/Taiwan/700TN1109a/2011/KT175076**^**a**^	Central America	~751	1271	207
	New Taipei City	DENV-1	I	D1/Taiwan/234NP1209a/2012/KT175078	Cambodia	4		
	Kaohsiung City	DENV-2	Cosmopolitan	D2/Taiwan/802KH1208a/2012/KT175112	Indonesia	~507		
	Kaohsiung City	DENV-3	III	D3/Taiwan/832KH1210a/2012/KP176715	Thailand	3		
	Kaohsiung City	DENV-4	II	D4/Taiwan/811KH1207a/2012/MG895380	Phillippine	4		
2013	Pingtung County	DENV-1	III	**D1/Taiwan/700TN1109a/2011/KT175076**^**a**^	Central America	36	596	264
	Pingtung County	DENV-2	Cosmopolitan	D2/Taiwan/920PT1306a/2013/KT175113	Indonesia	36		
	Southern Taiwan	DENV-2	Cosmopolitan	D2/Taiwan/900PT1308a/2013/KT175114	Indonesia	~500		
	Pingtung County	DENV-3	I	D3/Taiwan/932PT1305b/2013/MG895297	Indonesia	11		
2014	Kaohsiung City & Pingtung County	DENV-1	I	**D1/Taiwan/806KH1405a/2014/KU365900**^**b**^	Indonesia	~15490	15492	240
	Kaohsiung City	DENV-2	Asian 1	D2/Taiwan/807KH1411a/2014/KT175115	Myanmar	2		
2015	Kaohsiung City	DENV-1	I	**D1/Taiwan/806KH1405a/2014/KU365900**^**b**^	Indonesia	~163	43419	365
	Tainan City & Kaohsiung City	DENV-2	Cosmopolitan	**D2/Taiwan/704TN1505a/2015/KU365901**^**c**^	Indonesia	~43254		
	Tainan City	DENV-2	Cosmopolitan	D2/Taiwan/725TN1508a/2015/MG895173	India	2		
2016	Kaohsiung City	DENV-2	Cosmopolitan	**D2/Taiwan/704TN1505a/2015/KU365901**^**c**^	Indonesia	~372	380	363
	Taipei City	DENV-1	II	D1/Taiwan/114TP1611a/2016/KY496854	Philippines	3		

Boldface indicates the same epidemic strains circulated in Taiwan

^a^ between 2011 and 2013

^b^ between 2014 and 2015, and

^c^ between 2015 and 2016.

## Discussion

A total of 1,596 laboratory-confirmed imported dengue cases were identified in Taiwan during 2011–2016, most of which arrived from Asian countries and other regions, including the Pacific Islands, Australia, Africa and the Americas. Among them, 92.7% of cases (1,480 cases) arrived from the eight most common country sources of importation: Indonesia, the Philippines, Malaysia, Thailand, Vietnam, Myanmar, Singapore and Cambodia. An analysis of imported DENV strains from these countries showed changes in serotype distributions during the study period. As expected, all 4 serotypes of DENV were found to cocirculate in each of the most common country sources of importation during 2011–2016.

A total of 784 DENV strains, namely, 329 DENV-1, 227 DENV-2, 130 DENV-3, and 98 DENV-4 strains, were isolated during 2011–2016. Phylogenetic analyses of E gene sequences of imported DENV strains suggested that genotype I of DENV-1 and the Cosmopolitan genotype of DENV-2 were the predominant DENV strains imported from Southeast Asian countries during 2011–2016. Notably, genotype III of the DENV-1 strain was found to newly emerge in Malaysia, Vietnam, Thailand and Singapore. In addition, genotype III of the DENV-3 strain also emerged in Malaysia, Thailand and Singapore. However, Asian genotype 2 and the Asian/American genotype of the DENV-2 strain were not found in imported cases from Southeast Asian countries in the last decade, suggesting a low prevalence of these two genotypes in this region.

Previous studies have shown that DENV-1 genotype I was the predominant DENV genotype circulating in Southeast Asian countries [18, 19, 28–32]. In our study, the numbers of imported DENV-1 genotype I strains increased sharply in Indonesia and Malaysia and became the predominant genotype in the last decade. Phylogenetic analysis of E gene sequences of imported DENV-1 genotype I strains from Indonesia, Malaysia, Thailand and Vietnam showed a high degree of genetic diversity. Interestingly, we found that most of the DENV-1 genotype I strains did not segregate into a distinct clade in each country but that viral strains from Indonesia, Malaysia and a few strains from Singapore, Thailand, and Laos were clustered together. In addition, most of the imported DENV-1 genotype I strains from Thailand, Myanmar, Cambodia, China and Sri Lanka formed another cluster [33–35]. A DENV-1 genotype I strain (D1/Laos/1508aTw/2015) isolated from a case imported from Laos in 2015 is closely related to virus strains from Malaysia. Although only a few DENV strains have been isolated from imported cases from Laos in Taiwan during 2003–2016, genotype distribution of these imported DENV strains within each serotype is consistent with the results of a study by Castonguay-Vanier et al. [36]. Genotype III of DENV-1 strains imported from China, Vietnam, Malaysia, Singapore and Bangladesh were clustered together. The results suggest a close genetic relationship and frequent transmission of DENV-1 among Southeast Asian countries and may reflect frequent trade and travel between these countries [37, 38].

Genotype distribution of DENV-2 in Southeast Asian countries remains largely unchanged in the last decade. However, it is interesting to note that the number of DENV-2 Cosmopolitan genotype strains imported from Vietnam and Thailand increased and that Asian genotype I strains were found in Malaysia, indicating that these two genotypes of DENV-2 have expanded into new territories. Imported DENV-2 strains from the Maldives during 2015–2016 belonged to the Cosmopolitan genotype and are closely related to virus strains from Malaysia and Singapore. A DENV-2 Cosmopolitan genotype strain (D2/Palau/1612aTw/2016) isolated from a case imported from Palau is closely related to virus strains from the Philippines, Papua New Guinea and Fiji, suggesting cocirculation of these virus strains among these countries.

Except for Malaysia and Thailand, the genotype distribution of DENV-3 strains imported from Southeast Asian countries remains largely unchanged. It is interesting to note that the genotype of DENV-3 strains from Malaysia shifted from genotype I to genotype III. In addition, the genotype of DENV-3 strains from Thailand shifted from genotype II to genotype III in recent years. Recent studies have shown that the DENV-3 genotype III strains are emerging in Asian countries [39–41]. In this study, the number of DENV-3 genotype III strains has been increasing in Malaysia and Thailand, and these strains were clustered together with strains from Cambodia, Vietnam and Singapore.

Although a relatively low prevalence of DENV-4 strains was found in Southeast Asia [42], there was an increase in the numbers of DENV-4 strains imported from the Philippines, Myanmar and Cambodia, during 2011–2016. A DENV-4 genotype II strain (D4/Papua New Guinea/1608aTw/2016) isolated from a case imported from Papua New Guinea in 2016 is closely related to virus strains from Indonesia. Interestingly, we found that two DENV-1 genotype I strains (D1/Maldives/1604aTw/2016 and D1/Maldives/1608aTw/2016), a DENV-2 Cosmopolitan genotype strain (D2/Maldives/1612aTw/2016) and a DENV-4 genotype I strain (D4/Maldives/1605aTw/2016) were cocirculating in the Maldives in 2016, indicating multiple introductions of DENV strains into the Maldives in the recent dengue epidemic.

We previously reported that the genetic relationship and genotype distribution of DENV depend largely on the geographical location [18, 19]; however, our study demonstrates that geographical restrictions of DENV genotypes are becoming blurred. For example, DENV-1 genotype I, DENV-1 genotype III and DENV-2 Cosmopolitan genotype strains from different geographical locations or countries were clustered together, indicating the extensive introductions and continuous expansions of DENV strains between nations in Southeast Asia.

Due to the small sample size of imported cases from country sources of importation in Taiwan, the potential limitations of our study include: (1) Tourists/travelers may primarily travel to specific locations within a country, and thus, viruses circulating within other regions may not be represented often in our dataset; (2) Potentially changing patterns in travel (i.e. the number of travelers between Taiwan and country sources of importation and the purpose of travel) may affect the numbers of imported dengue cases and the representativeness of our analyses; (3) Because of the limited epidemiological data from country sources of importation, it is unclear whether the serotype/genotype dynamics described in this study are representative of the epidemiological situation within country.

Because air travel has become increasingly popular and convenient, DENV strains will be transmitted by travelers to other countries, sometimes leading to extensive outbreaks. During 2011–2016, several dengue outbreaks occurred in Taiwan, and most epidemic strains were introduced from neighboring Southeast Asian countries, including Indonesia, the Philippines, Vietnam and Myanmar (Table 2). In accordance with our previous studies, the patterns of imported DENV strains observed among the travelers are connected to the overall patterns of the dengue dynamics in Taiwan [19, 43]. It is worth noting that an epidemic DENV-1 strain (D1/Taiwan/700TN1109a/2011), which caused the dengue outbreak in southern Taiwan for three consecutive years (2011–2013), is closely related to virus strains from Central America. This is the first time that a dengue outbreak in Taiwan was caused by an American strain and suggests not only that imported DENV strains from neighboring Southeast Asian countries cause local outbreaks but also that viral strains introduced from the Americas or other continents may establish transmission chains within Taiwan.

In this study, we conducted molecular epidemiological analyses to monitor DENV serotype and genotype distributions and dynamic movements in Southeast Asian countries. The DENV strains isolated from imported dengue cases and the availability of a DENV genome sequence database can provide essential information on the global expansion and genetic evolution of DENV, which is useful for disease surveillance, laboratory diagnoses, pathogenesis investigation and vaccine development. Our results also indicate that it is important to reinforce active surveillance and travel and border health measures for dengue prevention and control in Taiwan.

## Supporting information

S1 Fig A phylogenetic tree of dengue virus type 1 (DENV-1). The phylogenetic tree is based on the complete E gene sequences of all DENV-1 isolates from imported and indigenous dengue cases in Taiwan during 2011–2016 and reference sequences obtained from GenBank. The tree was constructed by the neighbor-joining method and the maximum composite likelihood model. Bootstrap support values greater than 70 are shown. Viruses were identified by using the nomenclature of serotype/country/strain/year of isolation/GenBank accession number. The tree labels are colored by country, with Malaysia in dark red, Indonesia in green, Singapore in navy blue, China in purple, Thailand in deep yellow, the Philippines in blue green, Myanmar in red, Vietnam in peach, Cambodia in sky blue, Laos in light green, Australia in blue, Japan in yellow, and others and reference sequences in black color. The scale bar on the left indicates substitutions per site.
(TIF)

S2 Fig A phylogenetic tree of dengue virus type 2 (DENV-2). The phylogenetic tree is based on the complete E gene sequences of all DENV-2 isolates from imported and indigenous dengue cases in Taiwan during 2011–2016. See the legend of Supplementary Fig 1 for other details.
(TIF)

S3 Fig A phylogenetic tree of dengue virus type 3 (DENV-3). The phylogenetic tree is based on the complete E gene sequences of all DENV-3 isolates from imported and indigenous dengue cases in Taiwan during 2011–2016. See the legend of Supplementary Fig 1 for other details.
(TIF)

S4 Fig A phylogenetic tree of dengue virus type 4 (DENV-4). The phylogenetic tree is based on the complete E gene sequences of all DENV-4 isolates from imported and indigenous dengue cases in Taiwan during 2011–2016. See the legend of S1 Fig for other details.
(TIF)

S1 Table Strain identifiers and their accession numbers of imported and indigenous dengue virus strains in Taiwan during 2001–2006. (DOCX)
