# Supplementary figures and images for: Molecular characterization and phylogenetic analysis of dengue viruses imported into Taiwan during 2011-2016
Source: PLoS Negl Trop Dis. 2018 Sep 20;12(9):e0006773. doi: 10.1371/journal.pntd.0006773 (PMC6168156; doi:10.1371/journal.pntd.0006773)

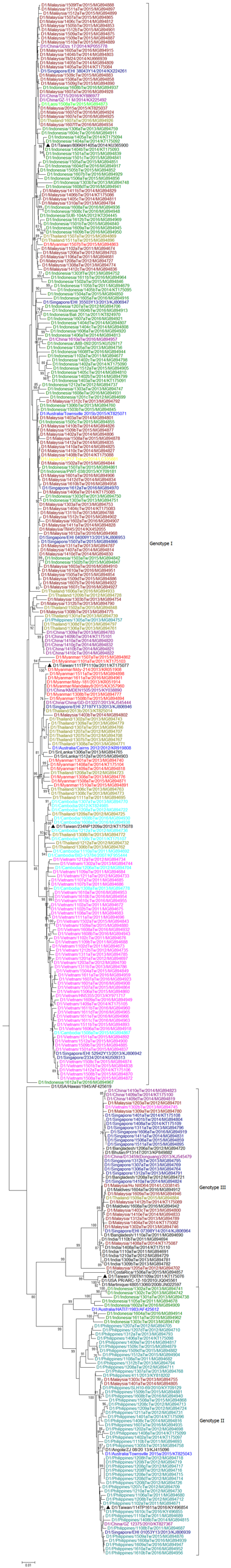

Supplement: S1 Fig — The phylogenetic tree is based on the complete E gene sequences of all DENV-1 isolates from imported and indigenous dengue cases in Taiwan during 2011–2016 and reference sequences obtained from GenBank. The tree was constructed by the neighbor-joining method and the maximum composite likelihood model. Bootstrap support values greater than 70 are shown. Viruses were identified by using the nomenclature of serotype/country/strain/year of isolation/GenBank accession number. The tree labels are colored by country, with Malaysia in dark red, Indonesia in green, Singapore in navy blue, China in purple, Thailand in deep yellow, the Philippines in blue green, Myanmar in red, Vietnam in peach, Cambodia in sky blue, Laos in light green, Australia in blue, Japan in yellow, and others and reference sequences in black color. The scale bar on the left indicates substitutions per site. (TIF) [file pntd.0006773.s001.tif]

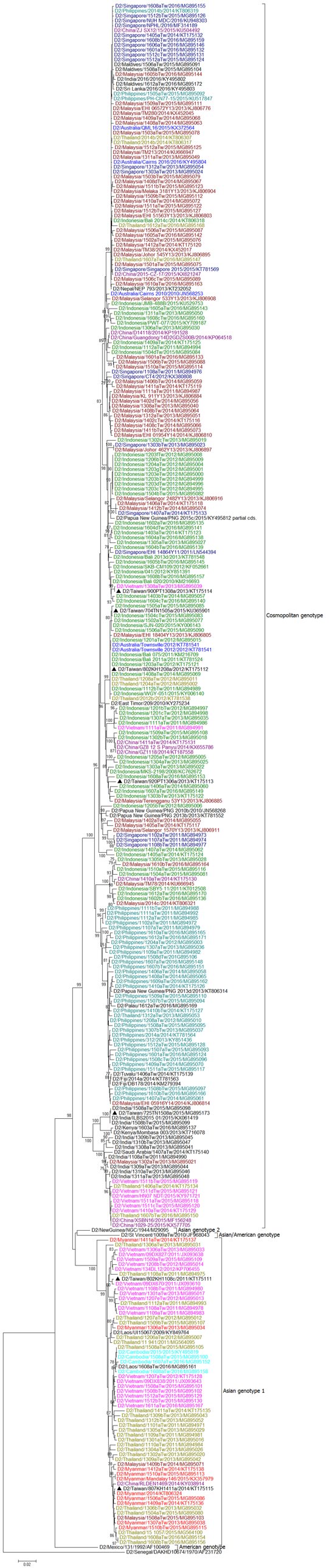

Supplement: S2 Fig — The phylogenetic tree is based on the complete E gene sequences of all DENV-2 isolates from imported and indigenous dengue cases in Taiwan during 2011–2016. See the legend of Supplementary Fig 1 for other details. (TIF) [file pntd.0006773.s002.tif]

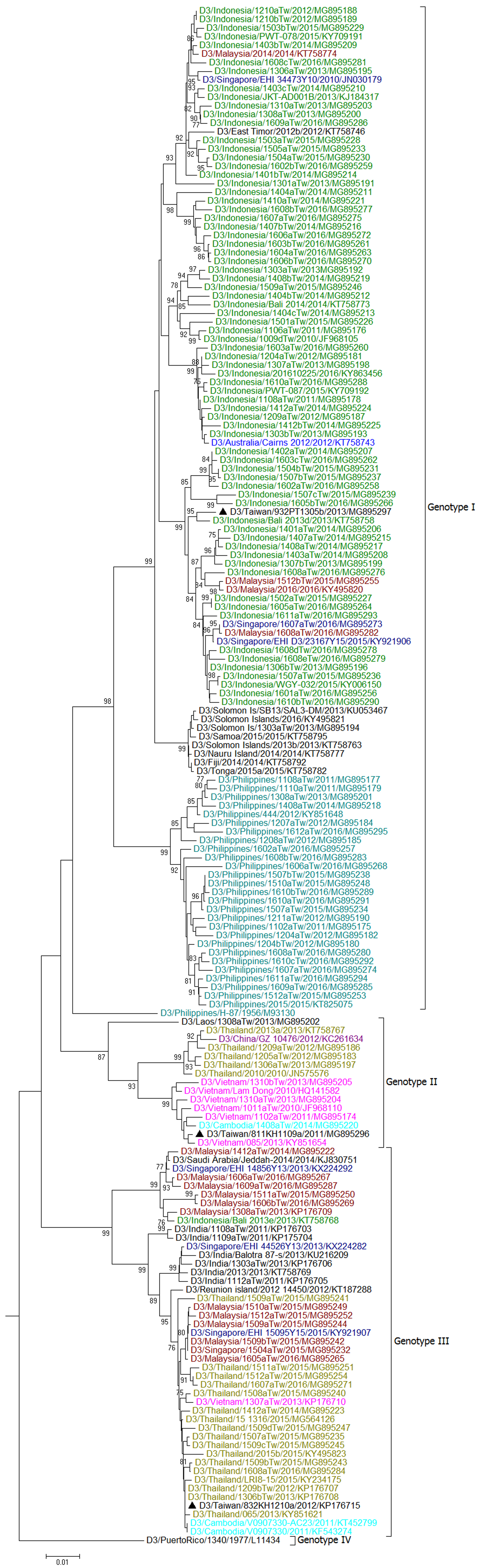

Supplement: S3 Fig — The phylogenetic tree is based on the complete E gene sequences of all DENV-3 isolates from imported and indigenous dengue cases in Taiwan during 2011–2016. See the legend of Supplementary Fig 1 for other details. (TIF) [file pntd.0006773.s003.tif]

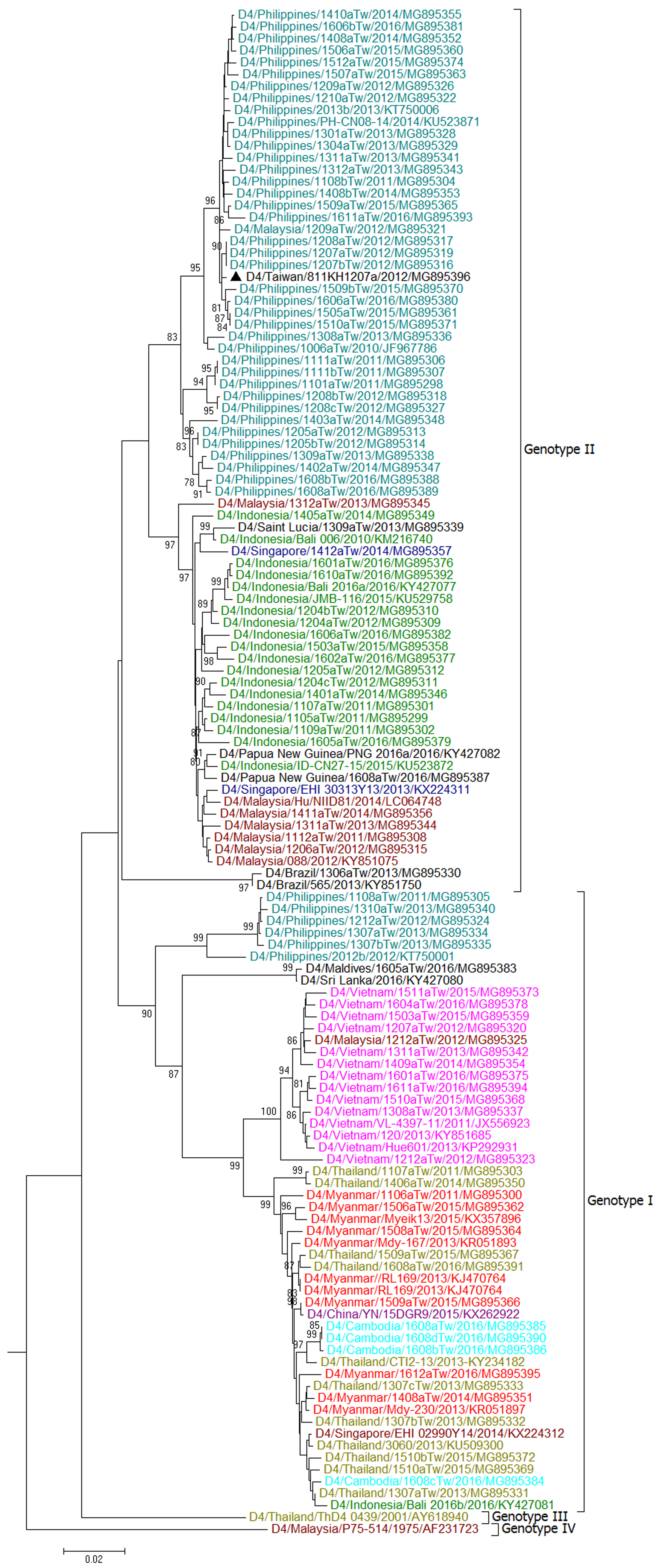

Supplement: S4 Fig — The phylogenetic tree is based on the complete E gene sequences of all DENV-4 isolates from imported and indigenous dengue cases in Taiwan during 2011–2016. See the legend of S1 Fig for other details. (TIF) [file pntd.0006773.s004.tif]
