# Supplementary material for: Molecular characterization and phylogenetic analysis of dengue viruses imported into Taiwan during 2011-2016
Source: PLoS Negl Trop Dis. 2018 Sep 20;12(9):e0006773. doi: 10.1371/journal.pntd.0006773 (PMC6168156; doi:10.1371/journal.pntd.0006773)
Supplement: S1 Table — (DOCX) [file pntd.0006773.s005.docx]

Table. Strain identifiers and their accession numbers of imported and indigenous dengue virus strains in Taiwan during 2001-2006.

| No. | Strain identifier | Gene | Sequence length | GenBank accession number |
| --- | --- | --- | --- | --- |
| 1 | D1/Taiwan/700TN1109a/2011 | E | 1485 | KT175076 |
| 2 | D1/Taiwan/111TP1110a/2011 | E | 1485 | KT175077 |
| 3 | D1/Taiwan/234NP1209a/2012 | E | 1485 | KT175078 |
| 4 | D1/Malaysia/1404aTw/2014 | E | 1485 | KT175082 |
| 5 | D1/Malaysia/1404cTw/2014 | E | 1485 | KT175083 |
| 6 | D1/Malaysia/1405aTw/2014 | E | 1485 | KT175084 |
| 7 | D1/Malaysia/1406aTw/2014 | E | 1485 | KT175085 |
| 8 | D1/Malaysia/1406bTw/2014 | E | 1485 | KT175086 |
| 9 | D1/Malaysia/1408aTw/2014 | E | 1485 | KT175087 |
| 10 | D1/Malaysia/1408bTw/2014 | E | 1485 | KT175088 |
| 11 | D1/Malaysia/1412bTw/2014 | E | 1485 | KT175089 |
| 12 | D1/Indonesia/1402aTw/2014 | E | 1485 | KT175090 |
| 13 | D1/Indonesia/1403aTw/2014 | E | 1485 | KT175091 |
| 14 | D1/Indonesia/1404aTw/2014 | E | 1485 | KT175092 |
| 15 | D1/Indonesia/1404bTw/2014 | E | 1485 | KT175093 |
| 16 | D1/Indonesia/1405aTw/2014 | E | 1485 | KT175094 |
| 17 | D1/Indonesia/1405bTw/2014 | E | 1485 | KT175095 |
| 18 | D1/Philippines/1401aTw/2014 | E | 1485 | KT175096 |
| 19 | D1/Philippines/1402aTw/2014 | E | 1485 | KT175097 |
| 20 | D1/Philippines/1406aTw/2014 | E | 1485 | KT175098 |
| 21 | D1/Philippines/1408aTw/2014 | E | 1485 | KT175099 |
| 22 | D1/China/1409aTw/2014 | E | 1485 | KT175100 |
| 23 | D1/China/1409bTw/2014 | E | 1485 | KT175101 |
| 24 | D1/Myanmar/1101aTw/2011 | E | 1485 | KT175103 |
| 25 | D1/Myanmar/1408aTw/2014 | E | 1485 | KT175104 |
| 26 | D1/Vietnam/1409aTw/2014 | E | 1485 | KT175105 |
| 27 | D1/Vietnam/1412aTw/2014 | E | 1485 | KT175106 |
| 28 | D1/Cambodia/1108cTw/2011 | E | 1485 | KT175107 |
| 29 | D1/Singapore/1401aTw/2014 | E | 1485 | KT175108 |
| 30 | D1/Singapore/1408aTw/2014 | E | 1485 | KT175109 |
| 31 | D1/India/1408aTw/2014 | E | 1485 | KT175110 |
| 32 | D1/Taiwan/806KH1405a/2014 | Complete cds | 10689 | KU365900 |
| 33 | D1/Taiwan/114TP1611a/2016 | Complete cds | 10689 | KY496854 |
| 34 | D1/Philippines/1610cTw/2016 | Complete cds | 10689 | KY496855 |
| 35 | D1/Philippines/1102aTw/2011 | E | 1485 | MG894671 |
| 36 | D1/Vietnam/1102aTw/2011 | E | 1485 | MG894672 |
| 37 | D1/Vietnam/1102dTw/2011 | E | 1485 | MG894673 |
| 38 | D1/Malaysia/1102aTw/2011 | E | 1485 | MG894674 |
| 39 | D1/Vietnam/1102bTw/2011 | E | 1485 | MG894675 |
| 40 | D1/Vietnam/1102cTw/2011 | E | 1485 | MG894676 |
| 41 | D1/Indonesia/1102aTw/2011 | E | 1485 | MG894677 |
| 42 | D1/Indonesia/1105aTw/2011 | E | 1485 | MG894678 |
| 43 | D1/Indonesia/1105bTw/2011 | E | 1485 | MG894679 |
| 44 | D1/Philippines/1106aTw/2011 | E | 1485 | MG894680 |
| 45 | D1/Malaysia/1106aTw/2011 | E | 1485 | MG894681 |
| 46 | D1/Philippines/1108aTw/2011 | E | 1485 | MG894682 |
| 47 | D1/Vietnam/1108aTw/2011 | E | 1485 | MG894683 |
| 48 | D1/Vietnam/1109aTw/2011 | E | 1485 | MG894684 |
| 49 | D1/Vietnam/1107aTw/2011 | E | 1485 | MG894685 |
| 50 | D1/Vietnam/1107bTw/2011 | E | 1485 | MG894686 |
| 51 | D1/Philippines/1108bTw/2011 | E | 1485 | MG894687 |
| 52 | D1/Vietnam/1109bTw/2011 | E | 1485 | MG894688 |
| 53 | D1/Philippines/1110aTw/2011 | E | 1485 | MG894689 |
| 54 | D1/Bangladesh/1110aTw/2011 | E | 1485 | MG894690 |
| 55 | D1/India/1110aTw/2011 | E | 1485 | MG894691 |
| 56 | D1/Cambodia/1110aTw/2011 | E | 1485 | MG894692 |
| 57 | D1/Philippines/1110bTw/2011 | E | 1485 | MG894693 |
| 58 | D1/India/1110bTw/2011 | E | 1485 | MG894694 |
| 59 | D1/Thailand/1111aTw/2011 | E | 1485 | MG894695 |
| 60 | D1/Vietnam/1111aTw/2011 | E | 1485 | MG894696 |
| 61 | D1/Vietnam/1201aTw/2012 | E | 1485 | MG894697 |
| 62 | D1/Philippines/1202aTw/2012 | E | 1485 | MG894698 |
| 63 | D1/Indonesia/1201cTw/2012 | E | 1485 | MG894699 |
| 64 | D1/Vietnam/1203aTw/2012 | E | 1485 | MG894700 |
| 65 | D1/Malaysia/1203aTw/2012 | E | 1485 | MG894701 |
| 66 | D1/Malaysia/1205aTw/2012 | E | 1485 | MG894702 |
| 67 | D1/Malaysia/1206aTw/2012 | E | 1485 | MG894703 |
| 68 | D1/Cambodia/1206aTw/2012 | E | 1485 | MG894704 |
| 69 | D1/Philippines/1207aTw/2012 | E | 1485 | MG894705 |
| 70 | D1/Indonesia/1207aTw/2012 | E | 1485 | MG894706 |
| 71 | D1/Thailand/1207aTw/2012 | E | 1485 | MG894707 |
| 72 | D1/Thailand/1207bTw/2012 | E | 1485 | MG894708 |
| 73 | D1/Philippines/1207cTw/2012 | E | 1485 | MG894709 |
| 74 | D1/Philippines/1207dTw/2012 | E | 1485 | MG894710 |
| 75 | D1/Philippines/1208aTw/2012 | E | 1485 | MG894711 |
| 76 | D1/Philippines/1208bTw/2012 | E | 1485 | MG894712 |
| 77 | D1/Philippines/1208cTw/2012 | E | 1485 | MG894713 |
| 78 | D1/Philippines/1208dTw/2012 | E | 1485 | MG894714 |
| 79 | D1/Philippines/1208eTw/2012 | E | 1485 | MG894715 |
| 80 | D1/Philippines/1208fTw/2012 | E | 1485 | MG894716 |
| 81 | D1/Philippines/1208gTw/2012 | E | 1485 | MG894717 |
| 82 | D1/Philippines/1208hTw/2012 | E | 1485 | MG894718 |
| 83 | D1/Philippines/1208iTw/2012 | E | 1485 | MG894719 |
| 84 | D1/Bangladesh/1206aTw/2012 | E | 1485 | MG894720 |
| 85 | D1/Bangladesh/1208aTw/2012 | E | 1485 | MG894721 |
| 86 | D1/Cambodia/1208aTw/2012 | E | 1485 | MG894722 |
| 87 | D1/Thailand/1208aTw/2012 | E | 1485 | MG894723 |
| 88 | D1/Philippines/1209aTw/2012 | E | 1485 | MG894724 |
| 89 | D1/Thailand/1209aTw/2012 | E | 1485 | MG894725 |
| 90 | D1/Philippines/1208jTw/2012 | E | 1485 | MG894726 |
| 91 | D1/Malaysia/1208aTw/2012 | E | 1485 | MG894727 |
| 92 | D1/Thailand/1209bTw/2012 | E | 1485 | MG894728 |
| 93 | D1/India/1210aTw/2012 | E | 1485 | MG894729 |
| 94 | D1/Philippines/1210aTw/2012 | E | 1485 | MG894730 |
| 95 | D1/Philippines/1211aTw/2012 | E | 1485 | MG894731 |
| 96 | D1/Thailand/1212aTw/2012 | E | 1485 | MG894732 |
| 97 | D1/Vietnam/1211aTw/2012 | E | 1485 | MG894733 |
| 98 | D1/Vietnam/1212aTw/2012 | E | 1485 | MG894734 |
| 99 | D1/Vietnam/1212bTw/2012 | E | 1485 | MG894735 |
| 100 | D1/Cambodia/1212aTw/2012 | E | 1485 | MG894736 |
| 101 | D1/Indonesia/1212aTw/2012 | E | 1485 | MG894737 |
| 102 | D1/Indonesia/1301aTw/2013 | E | 1485 | MG894738 |
| 103 | D1/Thailand/1301aTw/2013 | E | 1485 | MG894739 |
| 104 | D1/Myanmar/1301aTw/2013 | E | 1485 | MG894740 |
| 105 | D1/Indonesia/1302aTw/2013 | E | 1485 | MG894741 |
| 106 | D1/Indonesia/1302cTw/2013 | E | 1485 | MG894742 |
| 107 | D1/Thailand/1302aTw/2013 | E | 1485 | MG894743 |
| 108 | D1/Vietnam/1302aTw/2013 | E | 1485 | MG894744 |
| 109 | D1/Vietnam/1302bTw/2013 | E | 1485 | MG894745 |
| 110 | D1/Malaysia/1302aTw/2013 | E | 1485 | MG894746 |
| 111 | D1/Indonesia/1303aTw/2013 | E | 1485 | MG894747 |
| 112 | D1/Indonesia/1303bTw/2013 | E | 1485 | MG894748 |
| 113 | D1/Indonesia/1303cTw/2013 | E | 1485 | MG894749 |
| 114 | D1/Indonesia/1303dTw/2013 | E | 1485 | MG894750 |
| 115 | D1/Indonesia/1303eTw/2013 | E | 1485 | MG894751 |
| 116 | D1/Indonesia/1303fTw/2013 | E | 1485 | MG894752 |
| 117 | D1/Malaysia/1303aTw/2013 | E | 1485 | MG894753 |
| 118 | D1/Malaysia/1303bTw/2013 | E | 1485 | MG894754 |
| 119 | D1/Malaysia/1303cTw/2013 | E | 1485 | MG894755 |
| 120 | D1/Indonesia/1305aTw/2013 | E | 1485 | MG894756 |
| 121 | D1/Philippines/1305aTw/2013 | E | 1485 | MG894757 |
| 122 | D1/Philippines/1305bTw/2013 | E | 1485 | MG894758 |
| 123 | D1/Indonesia/1306aTw/2013 | E | 1485 | MG894759 |
| 124 | D1/Indonesia/1306bTw/2013 | E | 1485 | MG894760 |
| 125 | D1/Thailand/1306aTw/2013 | E | 1485 | MG894761 |
| 126 | D1/Thailand/1306bTw/2013 | E | 1485 | MG894762 |
| 127 | D1/Thailand/1306cTw/2013 | E | 1485 | MG894763 |
| 128 | D1/Singapore/1306aTw/2013 | E | 1485 | MG894764 |
| 129 | D1/SriLanka/1306aTw/2013 | E | 1485 | MG894765 |
| 130 | D1/Thailand/1307aTw/2013 | E | 1485 | MG894766 |
| 131 | D1/Thailand/1307bTw/2013 | E | 1485 | MG894767 |
| 132 | D1/Philippines/1307aTw/2013 | E | 1485 | MG894768 |
| 133 | D1/Singapore/1307aTw/2013 | E | 1485 | MG894769 |
| 134 | D1/Cambodia/1307aTw/2013 | E | 1485 | MG894770 |
| 135 | D1/Thailand/1308aTw/2013 | E | 1485 | MG894771 |
| 136 | D1/Thailand/1308bTw/2013 | E | 1485 | MG894772 |
| 137 | D1/Thailand/1308cTw/2013 | E | 1485 | MG894773 |
| 138 | D1/Malaysia/1308aTw/2013 | E | 1485 | MG894774 |
| 139 | D1/Malaysia/1308bTw/2013 | E | 1485 | MG894775 |
| 140 | D1/Myanmar/1308aTw/2013 | E | 1485 | MG894776 |
| 141 | D1/Myanmar/1308bTw/2013 | E | 1485 | MG894777 |
| 142 | D1/Cambodia/1308aTw/2013 | E | 1485 | MG894778 |
| 143 | D1/Thailand/1309aTw/2013 | E | 1485 | MG894779 |
| 144 | D1/Malaysia/1309aTw/2013 | E | 1485 | MG894780 |
| 145 | D1/India/1309aTw/2013 | E | 1485 | MG894781 |
| 146 | D1/India/1309bTw/2013 | E | 1485 | MG894782 |
| 147 | D1/China/1309aTw/2013 | E | 1485 | MG894783 |
| 148 | D1/Malaysia/1310aTw/2013 | E | 1485 | MG894784 |
| 149 | D1/Vietnam/1311aTw/2013 | E | 1485 | MG894785 |
| 150 | D1/Vietnam/1311bTw/2013 | E | 1485 | MG894786 |
| 151 | D1/Malaysia/1311aTw/2013 | E | 1485 | MG894787 |
| 152 | D1/Malaysia/1311bTw/2013 | E | 1485 | MG894788 |
| 153 | D1/Malaysia/1312aTw/2013 | E | 1485 | MG894789 |
| 154 | D1/Malaysia/1312bTw/2013 | E | 1485 | MG894790 |
| 155 | D1/Singapore/1312aTw/2013 | E | 1485 | MG894791 |
| 156 | D1/Malaysia/1312cTw/2013 | E | 1485 | MG894792 |
| 157 | D1/Philippines/1312aTw/2013 | E | 1485 | MG894793 |
| 158 | D1/Philippines/1312bTw/2013 | E | 1485 | MG894794 |
| 159 | D1/Singapore/1312bTw/2013 | E | 1485 | MG894795 |
| 160 | D1/Singapore/1311aTw/2013 | E | 1485 | MG894796 |
| 161 | D1/Thailand/1308dTw/2013 | E | 1485 | MG894797 |
| 162 | D1/Indonesia/1402cTw/2014 | E | 1485 | MG894798 |
| 163 | D1/Indonesia/1402bTw/2014 | E | 1485 | MG894799 |
| 164 | D1/Malaysia/1403cTw/2013 | E | 1485 | MG894800 |
| 165 | D1/Malaysia/1403aTw/2014 | E | 1485 | MG894801 |
| 166 | D1/Malaysia/1403bTw/2014 | E | 1485 | MG894802 |
| 167 | D1/Malaysia/1404bTw/2014 | E | 1485 | MG894803 |
| 168 | D1/Singapore/1401bTw/2014 | E | 1485 | MG894804 |
| 169 | D1/Malaysia/1401aTw/2014 | E | 1485 | MG894805 |
| 170 | D1/Malaysia/1402aTw/2014 | E | 1485 | MG894806 |
| 171 | D1/Indonesia/1404dTw/2014 | E | 1485 | MG894807 |
| 172 | D1/Indonesia/1404cTw/2014 | E | 1485 | MG894808 |
| 173 | D1/Malaysia/1405bTw/2014 | E | 1485 | MG894809 |
| 174 | D1/Indonesia/1405cTw/2014 | E | 1485 | MG894810 |
| 175 | D1/Malaysia/1405cTw/2014 | E | 1485 | MG894811 |
| 176 | D1/Malaysia/1406cTw/2014 | E | 1485 | MG894812 |
| 177 | D1/Indonesia/1406aTw/2014 | E | 1485 | MG894813 |
| 178 | D1/Malaysia/1407aTw/2014 | E | 1485 | MG894814 |
| 179 | D1/Philippines/1408bTw/2014 | E | 1485 | MG894815 |
| 180 | D1/Philippines/1408cTw/2014 | E | 1485 | MG894816 |
| 181 | D1/Philippines/1409aTw/2014 | E | 1485 | MG894817 |
| 182 | D1/Myanmar/1409aTw/2014 | E | 1485 | MG894818 |
| 183 | D1/China/1409dTw/2014 | E | 1485 | MG894819 |
| 184 | D1/China/1410aTw/2014 | E | 1485 | MG894820 |
| 185 | D1/China/1410bTw/2014 | E | 1485 | MG894821 |
| 186 | D1/China/1410cTw/2014 | E | 1485 | MG894822 |
| 187 | D1/China/1410eTw/2014 | E | 1485 | MG894823 |
| 188 | D1/Singapore/1410aTw/2014 | E | 1485 | MG894824 |
| 189 | D1/Malaysia/1410aTw/2014 | E | 1485 | MG894825 |
| 190 | D1/Malaysia/1410bTw/2014 | E | 1485 | MG894826 |
| 191 | D1/Malaysia/1410cTw/2014 | E | 1485 | MG894827 |
| 192 | D1/Malaysia/1411aTw/2014 | E | 1485 | MG894828 |
| 193 | D1/Malaysia/1411bTw/2014 | E | 1485 | MG894829 |
| 194 | D1/Malaysia/1410dTw/2014 | E | 1485 | MG894830 |
| 195 | D1/Singapore/1411aTw/2014 | E | 1485 | MG894831 |
| 196 | D1/China/1410gTw/2014 | E | 1485 | MG894832 |
| 197 | D1/Malaysia/1410eTw/2014 | E | 1485 | MG894833 |
| 198 | D1/Malaysia/1412dTw/2014 | E | 1485 | MG894834 |
| 199 | D1/Malaysia/1412aTw/2014 | E | 1485 | MG894835 |
| 200 | D1/Malaysia/1412cTw/2014 | E | 1485 | MG894836 |
| 201 | D1/Vietnam/1501aTw/2015 | E | 1485 | MG894837 |
| 202 | D1/Vietnam/1501bTw/2015 | E | 1485 | MG894838 |
| 203 | D1/Indonesia/1501aTw/2015 | E | 1485 | MG894839 |
| 204 | D1/Indonesia/1501bTw/2015 | E | 1485 | MG894840 |
| 205 | D1/Indonesia/1501cTw/2015 | E | 1485 | MG894841 |
| 206 | D1/Indonesia/1503aTw/2015 | E | 1485 | MG894842 |
| 207 | D1/Vietnam/1502aTw/2015 | E | 1485 | MG894843 |
| 208 | D1/Malaysia/1502aTw/2015 | E | 1485 | MG894844 |
| 209 | D1/Indonesia/1503bTw/2015 | E | 1485 | MG894845 |
| 210 | D1/Indonesia/1502aTw/2015 | E | 1485 | MG894846 |
| 211 | D1/Indonesia/1502bTw/2015 | E | 1485 | MG894847 |
| 212 | D1/Thailand/1502aTw/2015 | E | 1485 | MG894848 |
| 213 | D1/Vietnam/1504aTw/2015 | E | 1485 | MG894849 |
| 214 | D1/Indonesia/1504aTw/2015 | E | 1485 | MG894850 |
| 215 | D1/Indonesia/1505aTw/2015 | E | 1485 | MG894851 |
| 216 | D1/Indonesia/1505bTw/2015 | E | 1485 | MG894852 |
| 217 | D1/Malaysia/1505bTw/2015 | E | 1485 | MG894853 |
| 218 | D1/Malaysia/1505aTw/2015 | E | 1485 | MG894854 |
| 219 | D1/Indonesia/1505cTw/2015 | E | 1485 | MG894855 |
| 220 | D1/Indonesia/1506aTw/2015 | E | 1485 | MG894856 |
| 221 | D1/CostaRica/1506aTw/2015 | E | 1485 | MG894857 |
| 222 | D1/Malaysia/1506aTw/2015 | E | 1485 | MG894858 |
| 223 | D1/Singapore/1506aTw/2015 | E | 1485 | MG894859 |
| 224 | D1/Vietnam/1506aTw/2015 | E | 1485 | MG894860 |
| 225 | D1/Indonesia/1507aTw/2015 | E | 1485 | MG894861 |
| 226 | D1/Myanmar/1507aTw/2015 | E | 1485 | MG894862 |
| 227 | D1/Myanmar/1507bTw/2015 | E | 1485 | MG894863 |
| 228 | D1/Vietnam/1507aTw/2015 | E | 1485 | MG894864 |
| 229 | D1/Malaysia/1507aTw/2015 | E | 1485 | MG894865 |
| 230 | D1/Singapore/1507aTw/2015 | E | 1485 | MG894866 |
| 231 | D1/Cambodia/1508aTw/2015 | E | 1485 | MG894867 |
| 232 | D1/Philippines/1508aTw/2015 | E | 1485 | MG894868 |
| 233 | D1/Thailand/1507aTw/2015 | E | 1485 | MG894869 |
| 234 | D1/Vietnam/1508bTw/2015 | E | 1485 | MG894870 |
| 235 | D1/Myanmar/1508aTw/2015 | E | 1485 | MG894871 |
| 236 | D1/Vietnam/1508aTw/2015 | E | 1485 | MG894872 |
| 237 | D1/Laos/1508aTw/2015 | E | 1485 | MG894873 |
| 238 | D1/Vietnam/1508cTw/2015 | E | 1485 | MG894874 |
| 239 | D1/Malaysia/1509aTw/2015 | E | 1485 | MG894875 |
| 240 | D1/Philippines/1509aTw/2015 | E | 1485 | MG894876 |
| 241 | D1/Malaysia/1509bTw/2015 | E | 1485 | MG894877 |
| 242 | D1/Malaysia/1508aTw/2015 | E | 1485 | MG894878 |
| 243 | D1/Philippines/1509cTw/2015 | E | 1485 | MG894879 |
| 244 | D1/Vietnam/1509aTw/2015 | E | 1485 | MG894880 |
| 245 | D1/Philippines/1509bTw/2015 | E | 1485 | MG894881 |
| 246 | D1/Philippines/1509dTw/2015 | E | 1485 | MG894882 |
| 247 | D1/Malaysia/1509cTw/2015 | E | 1485 | MG894883 |
| 248 | D1/Thailand/1509aTw/2015 | E | 1485 | MG894884 |
| 249 | D1/Vietnam/1509bTw/2015 | E | 1485 | MG894885 |
| 250 | D1/Malaysia/1509dTw/2015 | E | 1485 | MG894886 |
| 251 | D1/Malaysia/1509eTw/2015 | E | 1485 | MG894887 |
| 252 | D1/Malaysia/1509fTw/2015 | E | 1485 | MG894888 |
| 253 | D1/Malaysia/1510aTw/2015 | E | 1485 | MG894889 |
| 254 | D1/Malaysia/1509gTw/2015 | E | 1485 | MG894890 |
| 255 | D1/Myanmar/1510aTw/2015 | E | 1485 | MG894891 |
| 256 | D1/Vietnam/1511aTw/2015 | E | 1485 | MG894892 |
| 257 | D1/Vietnam/1511bTw/2015 | E | 1485 | MG894893 |
| 258 | D1/Myanmar/1508bTw/2015 | E | 1485 | MG894894 |
| 259 | D1/Singapore/1511aTw/2015 | E | 1485 | MG894895 |
| 260 | D1/Thailand/1511aTw/2015 | E | 1485 | MG894896 |
| 261 | D1/Malaysia/1511aTw/2015 | E | 1485 | MG894897 |
| 262 | D1/Myanmar/1511aTw/2015 | E | 1485 | MG894898 |
| 263 | D1/Malaysia/1512aTw/2015 | E | 1485 | MG894899 |
| 264 | D1/Vietnam/1512aTw/2015 | E | 1485 | MG894900 |
| 265 | D1/Malaysia/1512bTw/2015 | E | 1485 | MG894901 |
| 266 | D1/Malaysia/1512cTw/2015 | E | 1485 | MG894902 |
| 267 | D1/SriLanka/1512aTw/2015 | E | 1485 | MG894903 |
| 268 | D1/Philippines/1512aTw/2015 | E | 1485 | MG894904 |
| 269 | D1/Indonesia/1512aTw/2015 | E | 1485 | MG894905 |
| 270 | D1/Malaysia/1601aTw/2016 | E | 1485 | MG894906 |
| 271 | D1/Malaysia/1602aTw/2016 | E | 1485 | MG894907 |
| 272 | D1/Vietnam/1601aTw/2016 | E | 1485 | MG894908 |
| 273 | D1/Indonesia/1602aTw/2016 | E | 1485 | MG894909 |
| 274 | D1/Malaysia/1603aTw/2016 | E | 1485 | MG894910 |
| 275 | D1/Indonesia/1604cTw/2016 | E | 1485 | MG894911 |
| 276 | D1/Maldives/1604aTw/2016 | E | 1485 | MG894912 |
| 277 | D1/Indonesia/1604bTw/2016 | E | 1485 | MG894913 |
| 278 | D1/Indonesia/1604aTw/2016 | E | 1485 | MG894914 |
| 279 | D1/Malaysia/1605aTw/2016 | E | 1485 | MG894915 |
| 280 | D1/Indonesia/1605aTw/2016 | E | 1485 | MG894916 |
| 281 | D1/Indonesia/1604dTw/2016 | E | 1485 | MG894917 |
| 282 | D1/Vietnam/1606aTw/2016 | E | 1485 | MG894918 |
| 283 | D1/Singapore/1606aTw/2016 | E | 1485 | MG894919 |
| 284 | D1/Indonesia/1606aTw/2016 | E | 1485 | MG894920 |
| 285 | D1/Indonesia/1607aTw/2016 | E | 1485 | MG894921 |
| 286 | D1/Malaysia/1607bTw/2016 | E | 1485 | MG894922 |
| 287 | D1/Vietnam/1607aTw/2016 | E | 1485 | MG894923 |
| 288 | D1/Malaysia/1607dTw/2016 | E | 1485 | MG894924 |
| 289 | D1/Malaysia/1607eTw/2016 | E | 1485 | MG894925 |
| 290 | D1/Thailand/1607aTw/2016 | E | 1485 | MG894926 |
| 291 | D1/Malaysia/1607cTw/2016 | E | 1485 | MG894927 |
| 292 | D1/Malaysia/1607aTw/2016 | E | 1485 | MG894928 |
| 293 | D1/Indonesia/1607bTw/2016 | E | 1485 | MG894929 |
| 294 | D1/Cambodia/1608bTw/2016 | E | 1485 | MG894930 |
| 295 | D1/Indonesia/1608eTw/2016 | E | 1485 | MG894931 |
| 296 | D1/Vietnam/1608aTw/2016 | E | 1485 | MG894932 |
| 297 | D1/Thailand/1606aTw/2016 | E | 1485 | MG894933 |
| 298 | D1/Malaysia/1607fTw/2016 | E | 1485 | MG894934 |
| 299 | D1/Philippines/1607aTw/2016 | E | 1485 | MG894935 |
| 300 | D1/Indonesia/1608aTw/2016 | E | 1485 | MG894936 |
| 301 | D1/Indonesia/1608bTw/2016 | E | 1485 | MG894937 |
| 302 | D1/Cambodia/1608aTw/2016 | E | 1485 | MG894938 |
| 303 | D1/Philippines/1608aTw/2016 | E | 1485 | MG894939 |
| 304 | D1/Philippines/1608bTw/2016 | E | 1485 | MG894940 |
| 305 | D1/Indonesia/1608dTw/2016 | E | 1485 | MG894941 |
| 306 | D1/Maldives/1608aTw/2016 | E | 1485 | MG894942 |
| 307 | D1/Vietnam/1608bTw/2016 | E | 1485 | MG894943 |
| 308 | D1/Indonesia/1608fTw/2016 | E | 1485 | MG894944 |
| 309 | D1/Indonesia/1609aTw/2016 | E | 1485 | MG894945 |
| 310 | D1/Malaysia/1609aTw/2016 | E | 1485 | MG894946 |
| 311 | D1/Philippines/1609aTw/2016 | E | 1485 | MG894947 |
| 312 | D1/Indonesia/1608cTw/2016 | E | 1485 | MG894948 |
| 313 | D1/Vietnam/1609aTw/2016 | E | 1485 | MG894949 |
| 314 | D1/Indonesia/1609bTw/2016 | E | 1485 | MG894950 |
| 315 | D1/Malaysia/1610aTw/2016 | E | 1485 | MG894951 |
| 316 | D1/Philippines/1610aTw/2016 | E | 1485 | MG894952 |
| 317 | D1/Vietnam/1610aTw/2016 | E | 1485 | MG894953 |
| 318 | D1/Vietnam/1610bTw/2016 | E | 1485 | MG894954 |
| 319 | D1/Vietnam/1610cTw/2016 | E | 1485 | MG894955 |
| 320 | D1/Philippines/1610bTw/2016 | E | 1485 | MG894956 |
| 321 | D1/China/1610aTw/2016 | E | 1485 | MG894957 |
| 322 | D1/Malaysia/1610bTw/2016 | E | 1485 | MG894958 |
| 323 | D1/Vietnam/1611aTw/2016 | E | 1485 | MG894959 |
| 324 | D1/Vietnam/1611bTw/2016 | E | 1485 | MG894960 |
| 325 | D1/Myanmar/1611aTw/2016 | E | 1485 | MG894961 |
| 326 | D1/Indonesia/1611aTw/2016 | E | 1485 | MG894962 |
| 327 | D1/Vietnam/1611cTw/2016 | E | 1485 | MG894963 |
| 328 | D1/Indonesia/1611bTw/2016 | E | 1485 | MG894964 |
| 329 | D1/Vietnam/1611dTw/2016 | E | 1485 | MG894965 |
| 330 | D1/Vietnam/1611eTw/2016 | E | 1485 | MG894966 |
| 331 | D1/Indonesia/1612aTw/2016 | E | 1485 | MG894967 |
| 332 | D1/Malaysia/1612aTw/2016 | E | 1485 | MG894968 |
| 333 | D1/Indonesia/1612bTw/2016 | E | 1485 | MG894969 |
| 334 | D1/Singapore/1612aTw/2016 | E | 1485 | MG894970 |
| 335 | D2/Taiwan/802KH1108c/2011 | E | 1485 | KT175111 |
| 336 | D2/Taiwan/802KH1208a/2012 | E | 1485 | KT175112 |
| 337 | D2/Taiwan/920PT1306a/2013 | E | 1485 | KT175113 |
| 338 | D2/Taiwan/900PT1308a/2013 | E | 1485 | KT175114 |
| 339 | D2/Taiwan/807KH1411a/2014 | E | 1485 | KT175115 |
| 340 | D2/Malaysia/1402cTw/2014 | E | 1485 | KT175116 |
| 341 | D2/Malaysia/1405aTw/2014 | E | 1485 | KT175117 |
| 342 | D2/Malaysia/1406aTw/2014 | E | 1485 | KT175118 |
| 343 | D2/Malaysia/1411aTw/2014 | E | 1485 | KT175119 |
| 344 | D2/Malaysia/1412aTw/2014 | E | 1485 | KT175120 |
| 345 | D2/Indonesia/1203aTw/2012 | E | 1485 | KT175121 |
| 346 | D2/Indonesia/1303bTw/2013 | E | 1485 | KT175122 |
| 347 | D2/Indonesia/1403aTw/2014 | E | 1485 | KT175123 |
| 348 | D2/Indonesia/1405aTw/2014 | E | 1485 | KT175124 |
| 349 | D2/Indonesia/1409aTw/2014 | E | 1485 | KT175125 |
| 350 | D2/Philippines/1410aTw/2014 | E | 1485 | KT175126 |
| 351 | D2/Philippines/1410bTw/2014 | E | 1485 | KT175127 |
| 352 | D2/Vietnam/1207aTw/2012 | E | 1485 | KT175128 |
| 353 | D2/Vietnam/1410aTw/2014 | E | 1485 | KT175129 |
| 354 | D2/China/1410aTw/2014 | E | 1485 | KT175130 |
| 355 | D2/China/1411aTw/2014 | E | 1485 | KT175131 |
| 356 | D2/Singapore/1405aTw/2014 | E | 1485 | KT175132 |
| 357 | D2/Singapore/1407aTw/2014 | E | 1485 | KT175133 |
| 358 | D2/Thailand/1406aTw/2014 | E | 1485 | KT175134 |
| 359 | D2/Thailand/1411aTw/2014 | E | 1485 | KT175135 |
| 360 | D2/Myanmar/1409aTw/2014 | E | 1485 | KT175136 |
| 361 | D2/Myanmar/1411aTw/2014 | E | 1485 | KT175137 |
| 362 | D2/Myanmar/1412aTw/2014 | E | 1485 | KT175138 |
| 363 | D2/Tuvalu/1406aTw/2014 | E | 1485 | KT175139 |
| 364 | D2/Saudi_Arabia/1407aTw/2014 | E | 1485 | KT175140 |
| 365 | D2/Taiwan/704TN1505a/2015 | Complete cds | 10638 | KU365901 |
| 366 | D2/Thailand/1101aTw/2011 | E | 1485 | MG894971 |
| 367 | D2/Philippines/1102aTw/2011 | E | 1485 | MG894972 |
| 368 | D2/Singapore/1102aTw/2011 | E | 1485 | MG894973 |
| 369 | D2/Singapore/1107aTw/2011 | E | 1485 | MG894974 |
| 370 | D2/Thailand/1108aTw/2011 | E | 1485 | MG894975 |
| 371 | D2/Singapore/1108aTw/2011 | E | 1485 | MG894976 |
| 372 | D2/Singapore/1108bTw/2011 | E | 1485 | MG894977 |
| 373 | D2/Vietnam/1108aTw/2011 | E | 1485 | MG894978 |
| 374 | D2/Philippines/1107aTw/2011 | E | 1485 | MG894979 |
| 375 | D2/Vietnam/1108bTw/2011 | E | 1485 | MG894980 |
| 376 | D2/Thailand/1109aTw/2011 | E | 1485 | MG894981 |
| 377 | D2/Philippines/1109aTw/2011 | E | 1485 | MG894982 |
| 378 | D2/Vietnam/1109aTw/2011 | E | 1485 | MG894983 |
| 379 | D2/Thailand/1110aTw/2011 | E | 1485 | MG894984 |
| 380 | D2/Philippines/1112aTw/2011 | E | 1485 | MG894985 |
| 381 | D2/Indonesia/1111aTw/2011 | E | 1485 | MG894986 |
| 382 | D2/Malaysia/1111aTw/2011 | E | 1485 | MG894987 |
| 383 | D2/Philippines/1111bTw/2011 | E | 1485 | MG894988 |
| 384 | D2/Indonesia/1112bTw/2011 | E | 1485 | MG894989 |
| 385 | D2/India/1108aTw/2011 | E | 1485 | MG894990 |
| 386 | D2/Vietnam/1111aTw/2011 | E | 1485 | MG894991 |
| 387 | D2/Philippines/1111aTw/2011 | E | 1485 | MG894992 |
| 388 | D2/Thailand/1112aTw/2011 | E | 1485 | MG894993 |
| 389 | D2/Indonesia/1112aTw/2011 | E | 1485 | MG894994 |
| 390 | D2/Indonesia/1203cTw/2012 | E | 1485 | MG894995 |
| 391 | D2/Indonesia/1203dTw/2012 | E | 1485 | MG894996 |
| 392 | D2/Indonesia/1201bTw/2012 | E | 1485 | MG894997 |
| 393 | D2/Indonesia/1201cTw/2012 | E | 1485 | MG894998 |
| 394 | D2/Indonesia/1203bTw/2012 | E | 1485 | MG894999 |
| 395 | D2/Indonesia/1203eTw/2012 | E | 1485 | MG895000 |
| 396 | D2/Indonesia/1203gTw/20012 | E | 1485 | MG895001 |
| 397 | D2/Thailand/1204aTw/2012 | E | 1485 | MG895002 |
| 398 | D2/Philippines/1204aTw/2012 | E | 1485 | MG895003 |
| 399 | D2/Indonesia/1204aTw/2012 | E | 1485 | MG895004 |
| 400 | D2/Indonesia/1205aTw/2012 | E | 1485 | MG895005 |
| 401 | D2/Indonesia/1205bTw/2012 | E | 1485 | MG895006 |
| 402 | D2/Thailand/1206aTw/2012 | E | 1485 | MG895007 |
| 403 | D2/Indonesia/1203fTw/2012 | E | 1485 | MG895008 |
| 404 | D2/Indonesia/1206bTw/2012 | E | 1485 | MG895009 |
| 405 | D2/Philippines/1208aTw/2012 | E | 1485 | MG895010 |
| 406 | D2/Thailand/1208aTw/2012 | E | 1485 | MG895011 |
| 407 | D2/Thailand/1207aTw/2012 | E | 1485 | MG895012 |
| 408 | D2/Vietnam/1207eTw/2012 | E | 1485 | MG895013 |
| 409 | D2/Vietnam/1208bTw/2012 | E | 1485 | MG895014 |
| 410 | D2/Indonesia/1201aTw/2012 | E | 1485 | MG895015 |
| 411 | D2/Thailand/1301aTw/2013 | E | 1485 | MG895016 |
| 412 | D2/Vietnam/1301aTw/2013 | E | 1485 | MG895017 |
| 413 | D2/Indonesia/1302bTw/2013 | E | 1485 | MG895018 |
| 414 | D2/Indonesia/1302cTw/2013 | E | 1485 | MG895019 |
| 415 | D2/Thailand/1302aTw/2013 | E | 1485 | MG895020 |
| 416 | D2/Malaysia/1302aTw/2013 | E | 1485 | MG895021 |
| 417 | D2/Indonesia/1303aTw/2013 | E | 1485 | MG895022 |
| 418 | D2/Singapore/1303bTw/2013 | E | 1485 | MG895023 |
| 419 | D2/Singapore/1303aTw/2013 | E | 1485 | MG895024 |
| 420 | D2/Indonesia/1304aTw/2013 | E | 1485 | MG895025 |
| 421 | D2/Thailand/1304aTw/2013 | E | 1485 | MG895026 |
| 422 | D2/Indonesia/1305aTw/2013 | E | 1485 | MG895027 |
| 423 | D2/Indonesia/1305bTw/2013 | E | 1485 | MG895028 |
| 424 | D2/Thailand/1305aTw/2013 | E | 1485 | MG895029 |
| 425 | D2/Indonesia/1306aTw/2013 | E | 1485 | MG895030 |
| 426 | D2/Thailand/1306aTw/2013 | E | 1485 | MG895031 |
| 427 | D2/Thailand/1306bTw/2013 | E | 1485 | MG895032 |
| 428 | D2/Vietnam/1306aTw/2013 | E | 1485 | MG895033 |
| 429 | D2/Myanmar/1306aTw/2013 | E | 1485 | MG895034 |
| 430 | D2/Indonesia/1307aTw/2013 | E | 1485 | MG895035 |
| 431 | D2/Philippines/1307aTw/2013 | E | 1485 | MG895036 |
| 432 | D2/Philippines/1307bTw/2013 | E | 1485 | MG895037 |
| 433 | D2/Myanmar/1307aTw/2013 | E | 1485 | MG895038 |
| 434 | D2/Vietnam/1308aTw/2013 | E | 1485 | MG895039 |
| 435 | D2/Malaysia/1308aTw/2013 | E | 1485 | MG895040 |
| 436 | D2/India/1308aTw/2013 | E | 1485 | MG895041 |
| 437 | D2/Thailand/1309aTw/2013 | E | 1485 | MG895042 |
| 438 | D2/Thailand/1309bTw/2013 | E | 1485 | MG895043 |
| 439 | D2/India/1309aTw/2013 | E | 1485 | MG895044 |
| 440 | D2/India/1309bTw/2013 | E | 1485 | MG895045 |
| 441 | D2/India/1310aTw/2013 | E | 1485 | MG895046 |
| 442 | D2/India/1310bTw/2013 | E | 1485 | MG895047 |
| 443 | D2/India/1311aTw/2013 | E | 1485 | MG895048 |
| 444 | D2/Malaysia/1311aTw/2013 | E | 1485 | MG895049 |
| 445 | D2/Indonesia/1311aTw/2013 | E | 1485 | MG895050 |
| 446 | D2/Malaysia/1312aTw/2013 | E | 1485 | MG895051 |
| 447 | D2/Thailand/1312bTw/2013 | E | 1485 | MG895052 |
| 448 | D2/Thailand/1312aTw/2013 | E | 1485 | MG895053 |
| 449 | D2/Singapore/1312aTw/2013 | E | 1485 | MG895054 |
| 450 | D2/Malaysia/1402aTw/2014 | E | 1485 | MG895055 |
| 451 | D2/Malaysia/1402dTw/2014 | E | 1485 | MG895056 |
| 452 | D2/Indonesia/1403bTw/2014 | E | 1485 | MG895057 |
| 453 | D2/Philippines/1406aTw/2014 | E | 1485 | MG895058 |
| 454 | D2/Malaysia/1406bTw/2014 | E | 1485 | MG895059 |
| 455 | D2/Indonesia/1406aTw/20014 | E | 1485 | MG895060 |
| 456 | D2/Philippines/1407aTw/2014 | E | 1485 | MG895061 |
| 457 | D2/Indonesia/1407aTw/2014 | E | 1485 | MG895062 |
| 458 | D2/Malaysia/1408aTw/2014 | E | 1485 | MG895063 |
| 459 | D2/Malaysia/1408bTw/2014 | E | 1485 | MG895064 |
| 460 | D2/Philippines/1408aTw/2014 | E | 1485 | MG895065 |
| 461 | D2/Malaysia/1408cTw/2014 | E | 1485 | MG895066 |
| 462 | D2/Malaysia/1408dTw/2014 | E | 1485 | MG895067 |
| 463 | D2/Malaysia/1409aTw/2014 | E | 1485 | MG895068 |
| 464 | D2/Indonesia/1408aTw/2014 | E | 1485 | MG895069 |
| 465 | D2/Philippines/1409aTw/2014 | E | 1485 | MG895070 |
| 466 | D2/Malaysia/1409bTw/2014 | E | 1485 | MG895071 |
| 467 | D2/Malaysia/1410aTw/2014 | E | 1485 | MG895072 |
| 468 | D2/Malaysia/1411bTw/2014 | E | 1485 | MG895073 |
| 469 | D2/Malaysia/1412bTw/2014 | E | 1485 | MG895074 |
| 470 | D2/Malaysia/1501aTw/2015 | E | 1485 | MG895075 |
| 471 | D2/Malaysia/1502aTw/2015 | E | 1485 | MG895076 |
| 472 | D2/Indonesia/1502aTw/2015 | E | 1485 | MG895077 |
| 473 | D2/Malaysia/1503aTw/2015 | E | 1485 | MG895078 |
| 474 | D2/Malaysia/1503bTw/2015 | E | 1485 | MG895079 |
| 475 | D2/Thailand/1504aTw/2015 | E | 1485 | MG895080 |
| 476 | D2/Indonesia/1504aTw/2015 | E | 1485 | MG895081 |
| 477 | D2/Indonesia/1504bTw/2015 | E | 1485 | MG895082 |
| 478 | D2/Indonesia/1504cTw/20015 | E | 1485 | MG895083 |
| 479 | D2/Indonesia/1504dTw/2015 | E | 1485 | MG895084 |
| 480 | D2/Indonesia/1505aTw/2015 | E | 1485 | MG895085 |
| 481 | D2/Myanmar/1506aTw/2015 | E | 1485 | MG895086 |
| 482 | D2/Malaysia/1506aTw/2015 | E | 1485 | MG895087 |
| 483 | D2/Malaysia/1506bTw/2015 | E | 1485 | MG895088 |
| 484 | D2/Malaysia/1506cTw/2015 | E | 1485 | MG895089 |
| 485 | D2/Indonesia/1506aTw/2015 | E | 1485 | MG895090 |
| 486 | D2/Maldives/1506aTw/2015 | E | 1485 | MG895091 |
| 487 | D2/Philippines/1505aTw/2015 | E | 1485 | MG895092 |
| 488 | D2/Philippines/1507aTw/2015 | E | 1485 | MG895093 |
| 489 | D2/Philippines/1507bTw/2015 | E | 1485 | MG895094 |
| 490 | D2/Philippines/1508aTw/2015 | E | 1485 | MG895095 |
| 491 | D2/Philippines/1508cTw/2015 | E | 1485 | MG895096 |
| 492 | D2/Philippines/1508bTw/2015 | E | 1485 | MG895097 |
| 493 | D2/India/1508aTw/2015 | E | 1485 | MG895098 |
| 494 | D2/India/1508bTw/2015 | E | 1485 | MG895099 |
| 495 | D2/Cambodia/1508aTw/2015 | E | 1485 | MG895100 |
| 496 | D2/Vietnam/1508aTw/2015 | E | 1485 | MG895101 |
| 497 | D2/Vietnam/1508bTw/2015 | E | 1485 | MG895102 |
| 498 | D2/Malaysia/1508aTw/2015 | E | 1485 | MG895103 |
| 499 | D2/Maldives/1508aTw/2015 | E | 1485 | MG895104 |
| 500 | D2/Thailand/1508aTw/2015 | E | 1485 | MG895105 |
| 501 | D2/Philippines/1508dTw/2015 | E | 1485 | MG895106 |
| 502 | D2/Thailand/1509bTw/2015 | E | 1485 | MG895107 |
| 503 | D2/Indonesia/1509aTw/2015 | E | 1485 | MG895108 |
| 504 | D2/Vietnam/1509aTw/2015 | E | 1485 | MG895109 |
| 505 | D2/Philippines/1509aTw/2015 | E | 1485 | MG895110 |
| 506 | D2/Malaysia/1509aTw/2015 | E | 1485 | MG895111 |
| 507 | D2/Malaysia/1509bTw/2015 | E | 1485 | MG895112 |
| 508 | D2/Myanmar/1510aTw/2015 | E | 1485 | MG895113 |
| 509 | D2/Malaysia/1510aTw/2015 | E | 1485 | MG895114 |
| 510 | D2/Myanmar/1510bTw/2015 | E | 1485 | MG895115 |
| 511 | D2/Indonesia/1510aTw/2015 | E | 1485 | MG895116 |
| 512 | D2/Philippines/1511aTw/2015 | E | 1485 | MG895117 |
| 513 | D2/Vietnam/1511aTw/2015 | E | 1485 | MG895118 |
| 514 | D2/Vietnam/1511bTw/2015 | E | 1485 | MG895119 |
| 515 | D2/Vietnam/1511cTw/2015 | E | 1485 | MG895120 |
| 516 | D2/Vietnam/1511dTw/2015 | E | 1485 | MG895121 |
| 517 | D2/Malaysia/1511aTw/2015 | E | 1485 | MG895122 |
| 518 | D2/Malaysia/1511bTw/2015 | E | 1485 | MG895123 |
| 519 | D2/Singapore/1512aTw/2015 | E | 1485 | MG895124 |
| 520 | D2/Malaysia/1512aTw/2015 | E | 1485 | MG895125 |
| 521 | D2/Singapore/1512bTw/2015 | E | 1485 | MG895126 |
| 522 | D2/Malaysia/1512bTw/2015 | E | 1485 | MG895127 |
| 523 | D2/Philippines/1512aTw/2015 | E | 1485 | MG895128 |
| 524 | D2/Vietnam/1512aTw/2015 | E | 1485 | MG895129 |
| 525 | D2/Vietnam/1512bTw/2015 | E | 1485 | MG895130 |
| 526 | D2/Singapore/1512cTw/2015 | E | 1485 | MG895131 |
| 527 | D2/Singapore/1601aTw/2016 | E | 1485 | MG895132 |
| 528 | D2/Malaysia/1601aTw/2016 | E | 1485 | MG895133 |
| 529 | D2/Philippines/1601aTw/2016 | E | 1485 | MG895134 |
| 530 | D2/Indonesia/1602aTw/2016 | E | 1485 | MG895135 |
| 531 | D2/Indonesia/1602bTw/2016 | E | 1485 | MG895136 |
| 532 | D2/Kenya/1603aTw/2016 | E | 1485 | MG895137 |
| 533 | D2/Indonesia/1604aTw/2016 | E | 1485 | MG895138 |
| 534 | D2/Indonesia/1604bTw/2016 | E | 1485 | MG895139 |
| 535 | D2/Indonesia/1604cTw/2016 | E | 1485 | MG895140 |
| 536 | D2/Indonesia/1604dTw/2016 | E | 1485 | MG895141 |
| 537 | D2/Malaysia/1605aTw/2016 | E | 1485 | MG895142 |
| 538 | D2/Indonesia/1605aTw/2016 | E | 1485 | MG895143 |
| 539 | D2/Malaysia/1605bTw/2016 | E | 1485 | MG895144 |
| 540 | D2/Indonesia/1605bTw/2016 | E | 1485 | MG895145 |
| 541 | D2/Singapore/1606aTw/2016 | E | 1485 | MG895146 |
| 542 | D2/Thailand/1607aTw/2016 | E | 1485 | MG895147 |
| 523 | D2/Philippines/1607aTw/2016 | E | 1485 | MG895148 |
| 544 | D2/Indonesia/1607aTw/2016 | E | 1485 | MG895149 |
| 545 | D2/Thailand/1607bTw/2016 | E | 1485 | MG895150 |
| 546 | D2/Philippines/1607bTw/2016 | E | 1485 | MG895151 |
| 547 | D2/Cambodia/1607aTw/2016 | E | 1485 | MG895152 |
| 548 | D2/Indonesia/1608aTw/2016 | E | 1485 | MG895153 |
| 549 | D2/Thailand/1608aTw/2016 | E | 1485 | MG895154 |
| 550 | D2/Singapore/1608aTw/2016 | E | 1485 | MG895155 |
| 551 | D2/Thailand/1608bTw/2016 | E | 1485 | MG895156 |
| 552 | D2/Indonesia/1608bTw/2016 | E | 1485 | MG895157 |
| 553 | D2/Cambodia/1608aTw/2016 | E | 1485 | MG895158 |
| 554 | D2/Singapore/1608bTw/2016 | E | 1485 | MG895159 |
| 555 | D2/Indonesia/1608cTw/2016 | E | 1485 | MG895160 |
| 556 | D2/Laos/1608aTw/2016 | E | 1485 | MG895161 |
| 557 | D2/Philippines/1609aTw/2016 | E | 1485 | MG895162 |
| 558 | D2/Malaysia/1610aTw/2016 | E | 1485 | MG895163 |
| 559 | D2/Malaysia/1610bTw/2016 | E | 1485 | MG895164 |
| 560 | D2/Philippines/1610aTw/2016 | E | 1485 | MG895165 |
| 561 | D2/Philippines/1610bTw/2016 | E | 1485 | MG895166 |
| 562 | D2/Vietnam/1611aTw/2016 | E | 1485 | MG895167 |
| 563 | D2/Thailand/1612aTw/2016 | E | 1485 | MG895168 |
| 564 | D2/Palau/1612aTw/2016 | E | 1485 | MG895169 |
| 565 | D2/Indonesia/1612aTw/2016 | E | 1485 | MG895170 |
| 566 | D2/Philippines/1612aTw/2016 | E | 1485 | MG895171 |
| 567 | D2/Maldives/1612aTw/2016 | E | 1485 | MG895172 |
| 568 | D2/Taiwan/725TN1508a/2015 | E | 1485 | MG895173 |
| 569 | D3/India/1108aTw/2011 | E | 1479 | KP176703 |
| 570 | D3/India/1109aTw/2011 | E | 1479 | KP176704 |
| 571 | D3/India/1112aTw/2011 | E | 1479 | KP176705 |
| 572 | D3/India/1303aTw/2013 | E | 1479 | KP176706 |
| 573 | D3/Thailand/1209bTw/2012 | E | 1479 | KP176707 |
| 574 | D3/Thailand/1306bTw/2013 | E | 1479 | KP176708 |
| 575 | D3/Malaysia/1308aTw/2013 | E | 1479 | KP176709 |
| 576 | D3/Vietnam/1307aTw/2013 | E | 1479 | KP176710 |
| 577 | D3/Taiwan/832KH1210a/2012 | E | 1479 | KP176715 |
| 578 | D3/Vietnam/1102aTw/2011 | E | 1479 | MG895174 |
| 579 | D3/Philippines/1102aTw/2011 | E | 1479 | MG895175 |
| 580 | D3/Indonesia/1106aTw/2011 | E | 1479 | MG895176 |
| 581 | D3/Philippines/1108aTw/2011 | E | 1479 | MG895177 |
| 582 | D3/Indonesia/1108aTw/2011 | E | 1479 | MG895178 |
| 583 | D3/Philippines/1110aTw/2011 | E | 1479 | MG895179 |
| 584 | D3/Philippines/1204bTw/2012 | E | 1479 | MG895180 |
| 585 | D3/Indonesia/1204aTw/2012 | E | 1479 | MG895181 |
| 586 | D3/Philippines/1204aTw/2012 | E | 1479 | MG895182 |
| 587 | D3/Thailand/1205aTw/2012 | E | 1479 | MG895183 |
| 588 | D3/Philippines/1207aTw/2012 | E | 1479 | MG895184 |
| 589 | D3/Philippines/1208aTw/2012 | E | 1479 | MG895185 |
| 590 | D3/Thailand/1209aTw/2012 | E | 1479 | MG895186 |
| 591 | D3/Indonesia/1209aTw/2012 | E | 1479 | MG895187 |
| 592 | D3/Indonesia/1210aTw/2012 | E | 1479 | MG895188 |
| 593 | D3/Indonesia/1210bTw/2012 | E | 1479 | MG895189 |
| 594 | D3/Philippines/1211aTw/2012 | E | 1479 | MG895190 |
| 595 | D3/Indonesia/1301aTw/2013 | E | 1479 | MG895191 |
| 596 | D3/Indonesia/1303aTw/2013 | E | 1479 | MG895192 |
| 597 | D3/Indonesia/1303bTw/2013 | E | 1479 | MG895193 |
| 598 | D3/Solomon_Is/1303aTw/2013 | E | 1479 | MG895194 |
| 599 | D3/Indonesia/1306aTw/2013 | E | 1479 | MG895195 |
| 600 | D3/Indonesia/1306bTw/2013 | E | 1479 | MG895196 |
| 601 | D3/Thailand/1306aTw/2013 | E | 1479 | MG895197 |
| 602 | D3/Indonesia/1307aTw/2013 | E | 1479 | MG895198 |
| 603 | D3/Indonesia/1307bTw/2013 | E | 1479 | MG895199 |
| 604 | D3/Indonesia/1308aTw/2013 | E | 1479 | MG895200 |
| 605 | D3/Philippines/1308aTw/2013 | E | 1479 | MG895201 |
| 606 | D3/Laos/1308aTw/2013 | E | 1479 | MG895202 |
| 607 | D3/Indonesia/1310aTw/2013 | E | 1479 | MG895203 |
| 608 | D3/Vietnam/1310aTw/2013 | E | 1479 | MG895204 |
| 609 | D3/Vietnam/1310bTw/2013 | E | 1479 | MG895205 |
| 610 | D3/Indonesia/1401aTw/2014 | E | 1479 | MG895206 |
| 611 | D3/Indonesia/1402aTw/2014 | E | 1479 | MG895207 |
| 612 | D3/Indonesia/1403aTw/2014 | E | 1479 | MG895208 |
| 613 | D3/Indonesia/1403bTw/2014 | E | 1479 | MG895209 |
| 614 | D3/Indonesia/1403cTw/2014 | E | 1479 | MG895210 |
| 615 | D3/Indonesia/1404aTw/2014 | E | 1479 | MG895211 |
| 616 | D3/Indonesia/1404bTw/2014 | E | 1479 | MG895212 |
| 617 | D3/Indonesia/1404cTw/2014 | E | 1479 | MG895213 |
| 618 | D3/Indonesia/1401bTw/2014 | E | 1479 | MG895214 |
| 619 | D3/Indonesia/1407aTw/2014 | E | 1479 | MG895215 |
| 620 | D3/Indonesia/1407bTw/2014 | E | 1479 | MG895216 |
| 621 | D3/Indonesia/1408aTw/2014 | E | 1479 | MG895217 |
| 622 | D3/Philippines/1408aTw/2014 | E | 1479 | MG895218 |
| 623 | D3/Indonesia/1408bTw/2014 | E | 1479 | MG895219 |
| 624 | D3/Cambodia/1408aTw/2014 | E | 1479 | MG895220 |
| 625 | D3/Indonesia/1410aTw/2014 | E | 1479 | MG895221 |
| 626 | D3/Malaysia/1412aTw/2014 | E | 1479 | MG895222 |
| 627 | D3/Thailand/1412aTw/2014 | E | 1479 | MG895223 |
| 628 | D3/Indonesia/1412aTw/2014 | E | 1479 | MG895224 |
| 629 | D3/Indonesia/1412bTw/2014 | E | 1479 | MG895225 |
| 630 | D3/Indonesia/1501aTw/2015 | E | 1479 | MG895226 |
| 631 | D3/Indonesia/1502aTw/2015 | E | 1479 | MG895227 |
| 632 | D3/Indonesia/1503aTw/2015 | E | 1479 | MG895228 |
| 633 | D3/Indonesia/1503bTw/2015 | E | 1479 | MG895229 |
| 634 | D3/Indonesia/1504aTw/2015 | E | 1479 | MG895230 |
| 635 | D3/Indonesia/1504bTw/2015 | E | 1479 | MG895231 |
| 636 | D3/Singapore/1504aTw/2015 | E | 1479 | MG895232 |
| 637 | D3/Indonesia/1505aTw/2015 | E | 1479 | MG895233 |
| 638 | D3/Philippines/1507aTw/2015 | E | 1479 | MG895234 |
| 639 | D3/Thailand/1507aTw/2015 | E | 1479 | MG895235 |
| 640 | D3/Indonesia/1507aTw/2015 | E | 1479 | MG895236 |
| 641 | D3/Indonesia/1507bTw/2015 | E | 1479 | MG895237 |
| 642 | D3/Philippines/1507bTw/2015 | E | 1479 | MG895238 |
| 643 | D3/Indonesia/1507cTw/2015 | E | 1479 | MG895239 |
| 644 | D3/Thailand/1508aTw/2015 | E | 1479 | MG895240 |
| 645 | D3/Thailand/1509aTw/2015 | E | 1479 | MG895241 |
| 646 | D3/Malaysia/1509bTw/2015 | E | 1479 | MG895242 |
| 647 | D3/Thailand/1509bTw/2015 | E | 1479 | MG895243 |
| 648 | D3/Malaysia/1509aTw/2015 | E | 1479 | MG895244 |
| 649 | D3/Thailand/1509cTw/2015 | E | 1479 | MG895245 |
| 650 | D3/Indonesia/1509aTw/2015 | E | 1479 | MG895246 |
| 651 | D3/Thailand/1509dTw/2015 | E | 1479 | MG895247 |
| 652 | D3/Philippines/1510aTw/2015 | E | 1479 | MG895248 |
| 653 | D3/Malaysia/1510aTw/2015 | E | 1479 | MG895249 |
| 654 | D3/Malaysia/1511aTw/2015 | E | 1479 | MG895250 |
| 655 | D3/Thailand/1511aTw/2015 | E | 1479 | MG895251 |
| 656 | D3/Malaysia/1512aTw/2015 | E | 1479 | MG895252 |
| 657 | D3/Philippines/1512aTw/2015 | E | 1479 | MG895253 |
| 658 | D3/Thailand/1512aTw/2015 | E | 1479 | MG895254 |
| 659 | D3/Malaysia/1512bTw/2015 | E | 1479 | MG895255 |
| 660 | D3/Indonesia/1601aTw/2016 | E | 1479 | MG895256 |
| 661 | D3/Philippines/1602aTw/2016 | E | 1479 | MG895257 |
| 662 | D3/Indonesia/1602aTw/2016 | E | 1479 | MG895258 |
| 663 | D3/Indonesia/1602bTw/2016 | E | 1479 | MG895259 |
| 664 | D3/Indonesia/1603aTw/2016 | E | 1479 | MG895260 |
| 665 | D3/Indonesia/1603bTw/2016 | E | 1479 | MG895261 |
| 666 | D3/Indonesia/1603cTw/2016 | E | 1479 | MG895262 |
| 667 | D3/Indonesia/1604aTw/2016 | E | 1479 | MG895263 |
| 668 | D3/Indonesia/1605aTw/2016 | E | 1479 | MG895264 |
| 669 | D3/Malaysia/1605aTw/2016 | E | 1479 | MG895265 |
| 670 | D3/Indonesia/1605bTw/2016 | E | 1479 | MG895266 |
| 671 | D3/Malaysia/1606aTw/2016 | E | 1479 | MG895267 |
| 672 | D3/Philippines/1606aTw/2016 | E | 1479 | MG895268 |
| 673 | D3/Malaysia/1606bTw/2016 | E | 1479 | MG895269 |
| 674 | D3/Indonesia/1606bTw/2016 | E | 1479 | MG895270 |
| 675 | D3/Thailand/1607aTw/2016 | E | 1479 | MG895271 |
| 676 | D3/Indonesia/1606aTw/2016 | E | 1479 | MG895272 |
| 677 | D3/Singapore/1607aTw/2016 | E | 1479 | MG895273 |
| 678 | D3/Philippines/1607aTw/2016 | E | 1479 | MG895274 |
| 679 | D3/Indonesia/1607aTw/2016 | E | 1479 | MG895275 |
| 680 | D3/Indonesia/1608aTw/2016 | E | 1479 | MG895276 |
| 681 | D3/Indonesia/1608bTw/2016 | E | 1479 | MG895277 |
| 682 | D3/Indonesia/1608dTw/2016 | E | 1479 | MG895278 |
| 683 | D3/Indonesia/1608eTw/2016 | E | 1479 | MG895279 |
| 684 | D3/Philippines/1608aTw/2016 | E | 1479 | MG895280 |
| 685 | D3/Indonesia/1608cTw/2016 | E | 1479 | MG895281 |
| 686 | D3/Malaysia/1608aTw/2016 | E | 1479 | MG895282 |
| 687 | D3/Philippines/1608bTw/2016 | E | 1479 | MG895283 |
| 688 | D3/Thailand/1608aTw/2016 | E | 1479 | MG895284 |
| 689 | D3/Philippines/1609aTw/2016 | E | 1479 | MG895285 |
| 690 | D3/Indonesia/1609aTw/2016 | E | 1479 | MG895286 |
| 691 | D3/Malaysia/1609aTw/2016 | E | 1479 | MG895287 |
| 692 | D3/Indonesia/1610aTw/2016 | E | 1479 | MG895288 |
| 693 | D3/Philippines/1610bTw/2016 | E | 1479 | MG895289 |
| 694 | D3/Indonesia/1610bTw/2016 | E | 1479 | MG895290 |
| 695 | D3/Philippines/1610aTw/2016 | E | 1479 | MG895291 |
| 696 | D3/Philippines/1610cTw/2016 | E | 1479 | MG895292 |
| 697 | D3/Indonesia/1611aTw/2016 | E | 1479 | MG895293 |
| 698 | D3/Philippines/1611aTw/2016 | E | 1479 | MG895294 |
| 699 | D3/Philippines/1612aTw/2016 | E | 1479 | MG895295 |
| 700 | D3/Taiwan/811KH1109a/2011 | E | 1479 | MG895296 |
| 701 | D3/Taiwan/932PT1305b/2013 | E | 1479 | MG895297 |
| 702 | D4/Philippines/1101aTw/2011 | E | 1485 | MG895298 |
| 703 | D4/Indonesia/1105aTw/2011 | E | 1485 | MG895299 |
| 704 | D4/Myanmar/1106aTw/2011 | E | 1485 | MG895300 |
| 705 | D4/Indonesia/1107aTw/2011 | E | 1485 | MG895301 |
| 706 | D4/Indonesia/1109aTw/2011 | E | 1485 | MG895302 |
| 707 | D4/Thailand/1107aTw/2011 | E | 1485 | MG895303 |
| 708 | D4/Philippines/1108bTw/2011 | E | 1485 | MG895304 |
| 709 | D4/Philippines/1108aTw/2011 | E | 1485 | MG895305 |
| 710 | D4/Philippines/1111aTw/2011 | E | 1485 | MG895306 |
| 711 | D4/Philippines/1111bTw/2011 | E | 1485 | MG895307 |
| 712 | D4/Malaysia/1112aTw/2011 | E | 1485 | MG895308 |
| 713 | D4/Indonesia/1204aTw/2012 | E | 1485 | MG895309 |
| 714 | D4/Indonesia/1204bTw/2012 | E | 1485 | MG895310 |
| 715 | D4/Indonesia/1204cTw/2012 | E | 1485 | MG895311 |
| 716 | D4/Indonesia/1205aTw/2012 | E | 1485 | MG895312 |
| 717 | D4/Philippines/1205aTw/2012 | E | 1485 | MG895313 |
| 718 | D4/Philippines/1205bTw/2012 | E | 1485 | MG895314 |
| 719 | D4/Malaysia/1206aTw/2012 | E | 1485 | MG895315 |
| 720 | D4/Philippines/1207bTw/2012 | E | 1485 | MG895316 |
| 721 | D4/Philippines/1208aTw/2012 | E | 1485 | MG895317 |
| 722 | D4/Philippines/1208bTw/2012 | E | 1485 | MG895318 |
| 723 | D4/Philippines/1207aTw/2012 | E | 1485 | MG895319 |
| 724 | D4/Vietnam/1207aTw/2012 | E | 1485 | MG895320 |
| 725 | D4/Malaysia/1209aTw/2012 | E | 1485 | MG895321 |
| 726 | D4/Philippines/1210aTw/2012 | E | 1485 | MG895322 |
| 727 | D4/Vietnam/1212aTw/2012 | E | 1485 | MG895323 |
| 728 | D4/Philippines/1212aTw/2012 | E | 1485 | MG895324 |
| 729 | D4/Malaysia/1212aTw/2012 | E | 1485 | MG895325 |
| 730 | D4/Philippines/1209aTw/2012 | E | 1485 | MG895326 |
| 731 | D4/Philippines/1208cTw/2012 | E | 1485 | MG895327 |
| 732 | D4/Philippines/1301aTw/2013 | E | 1485 | MG895328 |
| 733 | D4/Philippines/1304aTw/2013 | E | 1485 | MG895329 |
| 734 | D4/Brazil/1306aTw/2013 | E | 1485 | MG895330 |
| 735 | D4/Thailand/1307aTw/2013 | E | 1485 | MG895331 |
| 736 | D4/Thailand/1307bTw/2013 | E | 1485 | MG895332 |
| 737 | D4/Thailand/1307cTw/2013 | E | 1485 | MG895333 |
| 738 | D4/Philippines/1307aTw/2013 | E | 1485 | MG895334 |
| 739 | D4/Philippines/1307bTw/2013 | E | 1485 | MG895335 |
| 740 | D4/Philippines/1308aTw/2013 | E | 1485 | MG895336 |
| 741 | D4/Vietnam/1308aTw/2013 | E | 1485 | MG895337 |
| 742 | D4/Philippines/1309aTw/2013 | E | 1485 | MG895338 |
| 743 | D4/Saint_Lucia/1309aTw/2013 | E | 1485 | MG895339 |
| 744 | D4/Philippines/1310aTw/2013 | E | 1485 | MG895340 |
| 745 | D4/Philippines/1311aTw/2013 | E | 1485 | MG895341 |
| 746 | D4/Vietnam/1311aTw/2013 | E | 1485 | MG895342 |
| 747 | D4/Philippines/1312aTw/2013 | E | 1485 | MG895343 |
| 748 | D4/Malaysia/1311aTw/2013 | E | 1485 | MG895344 |
| 749 | D4/Malaysia/1312aTw/2013 | E | 1485 | MG895345 |
| 750 | D4/Indonesia/1401aTw/2014 | E | 1485 | MG895346 |
| 751 | D4/Philippines/1402aTw/2014 | E | 1485 | MG895347 |
| 752 | D4/Philippines/1403aTw/2014 | E | 1485 | MG895348 |
| 753 | D4/Indonesia/1405aTw/2014 | E | 1485 | MG895349 |
| 754 | D4/Thailand/1406aTw/2014 | E | 1485 | MG895350 |
| 755 | D4/Myanmar/1408aTw/2014 | E | 1485 | MG895351 |
| 756 | D4/Philippines/1408aTw/2014 | E | 1485 | MG895352 |
| 757 | D4/Philippines/1408bTw/2014 | E | 1485 | MG895353 |
| 758 | D4/Vietnam/1409aTw/2014 | E | 1485 | MG895354 |
| 759 | D4/Philippines/1410aTw/2014 | E | 1485 | MG895355 |
| 760 | D4/Malaysia/1411aTw/2014 | E | 1485 | MG895356 |
| 761 | D4/Singapore/1412aTw/2014 | E | 1485 | MG895357 |
| 762 | D4/Indonesia/1503aTw/2015 | E | 1485 | MG895358 |
| 763 | D4/Vietnam/1503aTw/2015 | E | 1485 | MG895359 |
| 764 | D4/Philippines/1506aTw/2015 | E | 1485 | MG895360 |
| 765 | D4/Philippines/1505aTw/2015 | E | 1485 | MG895361 |
| 766 | D4/Myanmar/1506aTw/2015 | E | 1485 | MG895362 |
| 767 | D4/Philippines/1507aTw/2015 | E | 1485 | MG895363 |
| 768 | D4/Myanmar/1508aTw/2015 | E | 1485 | MG895364 |
| 769 | D4/Philippines/1509aTw/2015 | E | 1485 | MG895365 |
| 770 | D4/Myanmar/1509aTw/2015 | E | 1485 | MG895366 |
| 771 | D4/Thailand/1509aTw/2015 | E | 1485 | MG895367 |
| 772 | D4/Vietnam/1510aTw/2015 | E | 1485 | MG895368 |
| 773 | D4/Thailand/1510aTw/2015 | E | 1485 | MG895369 |
| 774 | D4/Philippines/1509bTw/2015 | E | 1485 | MG895370 |
| 775 | D4/Philippines/1510aTw/2015 | E | 1485 | MG895371 |
| 776 | D4/Thailand/1510bTw/2015 | E | 1485 | MG895372 |
| 777 | D4/Vietnam/1511aTw/2015 | E | 1485 | MG895373 |
| 778 | D4/Philippines/1512aTw/2015 | E | 1485 | MG895374 |
| 779 | D4/Vietnam/1601aTw/2016 | E | 1485 | MG895375 |
| 780 | D4/Indonesia/1601aTw/2016 | E | 1485 | MG895376 |
| 781 | D4/Indonesia/1602aTw/2016 | E | 1485 | MG895377 |
| 782 | D4/Vietnam/1604aTw/2016 | E | 1485 | MG895378 |
| 783 | D4/Indonesia/1605aTw/2016 | E | 1485 | MG895379 |
| 784 | D4/Philippines/1606aTw/2016 | E | 1485 | MG895380 |
| 785 | D4/Philippines/1606bTw/2016 | E | 1485 | MG895381 |
| 786 | D4/Indonesia/1606aTw/2016 | E | 1485 | MG895382 |
| 787 | D4/Maldives/1605aTw/2016 | E | 1485 | MG895383 |
| 788 | D4/Cambodia/1608cTw/2016 | E | 1485 | MG895384 |
| 789 | D4/Cambodia/1608aTw/2016 | E | 1485 | MG895385 |
| 790 | D4/Cambodia/1608bTw/2016 | E | 1485 | MG895386 |
| 791 | D4/Papua_New_Guinea/1608aTw/2016 | E | 1485 | MG895387 |
| 792 | D4/Philippines/1608bTw/2016 | E | 1485 | MG895388 |
| 793 | D4/Philippines/1608aTw/2016 | E | 1485 | MG895389 |
| 794 | D4/Cambodia/1608dTw/2016 | E | 1485 | MG895390 |
| 795 | D4/Thailand/1608aTw/2016 | E | 1485 | MG895391 |
| 796 | D4/Indonesia/1610aTw/2016 | E | 1485 | MG895392 |
| 797 | D4/Philippines/1611aTw/2016 | E | 1485 | MG895393 |
| 798 | D4/Vietnam/1611aTw/2016 | E | 1485 | MG895394 |
| 799 | D4/Myanmar/1612aTw/2016 | E | 1485 | MG895395 |
| 800 | D4/Taiwan/811KH1207a/2012 | E | 1485 | MG895396 |

E: envelope gene. Complete cds: complete coding sequence of the polyprotein gene.
